# Distinct GmASMTs are involved in regulating transcription factors and signalling cross-talk across embryo development, biotic, and abiotic stress in soybean

**DOI:** 10.3389/fpls.2022.948901

**Published:** 2022-08-11

**Authors:** Gyanendra Kumar, Monisha Arya, Radhika Padma, Bijesh Puthusseri, Parvatam Giridhar

**Affiliations:** ^1^Plant Cell Biotechnology Department, CSIR-Central Food Technological Research Institute, Mysore, Karnataka, India; ^2^Indian Institute of Science Education and Research, Bhopal, Madhya Pradesh, India

**Keywords:** abiotic stress, auxin transport, biotic stress, circadian cycle, soybean, stress regulation, transcription factors, GWAS

## Abstract

N-Acetylserotonin O-methyltransferase (ASMT) is the final enzyme involved in melatonin biosynthesis. Identifying the expression of ASMT will reveal the regulatory role in the development and stress conditions in soybean. To identify and characterize ASMT in soybean (GmASMT), we employed genome-wide analysis, gene structure, cis-acting elements, gene expression, co-expression network analysis, and enzyme assay. We found seven pairs of segmental and tandem duplication pairs among the 44 identified GmASMTs by genome-wide analysis. Notably, co-expression network analysis reported that distinct GmASMTs are involved in various stress response. For example, GmASMT3, GmASMT44, GmASMT17, and GmASMT7 are involved in embryo development, heat, drought, aphid, and soybean cyst nematode infections, respectively. These distinct networks of GmASMTs were associated with transcription factors (NAC, MYB, WRKY, and ERF), stress signalling, isoflavone and secondary metabolites, calcium, and calmodulin proteins involved in stress regulation. Further, GmASMTs demonstrated auxin-like activities by regulating the genes involved in auxin transporter (WAT1 and NRT1/PTR) and auxin-responsive protein during developmental and biotic stress. The current study identified the key regulatory role of GmASMTs during development and stress. Hence GmASMT could be the primary target in genetic engineering for crop improvement under changing environmental conditions.

## Introduction

Melatonin (MEL) was first reported in plants and is widely distributed in vegetative and reproductive tissue (Dubbels et al., 1995; Hattori et al., 1995). They play an important role in circadian rhythm, biochemical and physiological processes in plants (Murch and Erland, 2021). They act as effective signalling and bioactive molecule associated with plants tolerance against abiotic and biotic stresses (Tan et al., 2019; Qari et al., 2022). Furthermore, they are associated with an increase in reactive oxygen and nitrogen species and protect plants by scavenging free radicals by regulating the expression of redox enzymes (Arnao and Hernández-Ruiz, 2019; Corpas et al., 2022). Additionally, it modulates the plant growth hormone cross-talk between auxin, abscisic acid, ethylene, gibberellic acid, methyl jasmonate, and salicylic acid to bring cellular and physiological changes to plants (Arnao and Hernández-Ruiz, 2018; Kumar et al., 2021b; Wang et al., 2022).

The final two steps in MEL biosynthesis in plants are carried out by serotonin N-acetyltransferase (SNAT) and N-Acetylserotonin O-methyltransferase (ASMT). Where serotonin (SER) is converted to N-Acetylserotonin by SNAT and methylated by ASMT to form MEL (Arnao and Hernández-Ruiz, 2018). Alternatively, SER can be methylated to 5-methoxytryptamine by AMST; after that, it is acetylated by SNAT to produce MEL. These two pathways are likely to occur in plants, animals, and microorganisms (Zhao et al., 2019). The different homologs of ASMT and SNAT have been identified between plants and animals, showing different origins during evolution (Zhao et al., 2019, 2021). The phylogenetic analysis suggested that ASMT emerged in primitive bacteria (cyanobacteria) and gradually expanded to terrestrial plants (Zhao et al., 2021).

ASMT and SNAT enzymes are critical for regulating MEL levels in plants (Pan et al., 2019). For example, the expression of ASMT is reported to increase dramatically in various abiotic and biotic stresses in *Oryza sativa* (Kang et al., 2011). Additionally, overexpression of ASMT is directly associated with the MEL level in *Malus zumi* and *Arabidopsis thaliana* (Zuo et al., 2014; Zhu et al., 2021). Previous studies have identified, cloned, and over-expressed ASMT from *A. thaliana* and *O. sativa* (Kang et al., 2011; Byeon et al., 2016). However, there are no reports about MEL and ASMT in *Glycine max* (soybean).

Soybean is one of the important economic crops grown majorly in the United States, accounting for 36.2 million harvested hectares and serves as an oilseed crop, protein source and biofuel feedstock (Assefa et al., 2018). The production and seed quality of soybean is significantly affected by environmental conditions. An efficient way of increasing abiotic and biotic stress tolerance is by identifying the key regulatory mechanism of the gene involved in stress regulation in soybean (Xu et al., 2018). Thus it is crucial to identify the key gene family which plays a vital role in development and stress tolerance. ASMT is an essential enzyme in plants MEL biosynthesis and biotic and abiotic stress regulation. Therefore, it could be the primary target for genetic engineering and crop improvement under changing environmental conditions.

Thus our study is aimed to identify the ASMTs through various means, and we have analysed GmASMTs cis-regulatory elements and expression datasets, including circadian, embryo development, abiotic and biotic stress, to explain the functions involved in various stress responses. Additionally, we discovered distinct regulatory networks acting in the development, and abiotic and biotic stress responses, through weighted gene co-expression network analysis (WGCNA). We also checked the involvement of SER/MEL during soybean seed germination, where the seeds were primed with various concentrations of SER and MEL. Further, GmASMTs were confirmed by cloning, molecular docking, and enzyme assay.

This is the first study reporting distinct ASMT networks in soybean development, biotic, and abiotic stress response. Our analyses identified the key regulatory role of GmASMTs during development and stress. Hence GmASMT could be the primary target in genetic engineering to achieve crop improvement under changing environmental conditions.

## Materials and methods

### Identification of the ASMT family

To identify the soybean ASMT candidates, the OsASMT and AtASMT were used as a query sequence to run the Hidden Markov Model (E-value <1e^−5^) to profile all the ASMTs from the soybean database. All the candidate sequences were analysed via PFAMScan^1^ and SMART^2^ to detect the presence of the ASMT domain. Molecular weight (MW), and isoelectric point (pI) of protein sequences were calculated using the ExPASY^3^ and conserved domains within GmASMT through NCBI conserved domain database (CDD).^4^

### Chromosomal location and gene duplication analysis

The soybean GmASMTs was determined based on physical positions on chromosomes corresponding to their locus numbers present in the EnsemblPlants database.^5^ The duplication of GmASMTs was aligned using BLASTP for the protein sequence with an e-value of e^−10^ and MCScanX to classify the duplication patterns, including segmental duplications, and are represented using Circos (Chen et al., 2020).

### Phylogeny analysis

The protein sequences of all the putative GmASMTs were aligned in MEGAX using MUSCLE. We performed phylogenetic analyses based on the maximum likelihood method using MEGAX. The Non-synonymous (Ka) to synonymous (Ks) substitution was used to assess selection history and divergence time. The Ks and Ka substitutions of tandem/segmental duplicated gene pairs of GmASMTs were calculated using the Ka/Ks Calculator 2.0 program.^6^ The divergence time (T) was calculated using the formula T = Ks / (2 × 6.1 × 10^−9^) × 10^−6^ million years ago (Ahmad et al., 2019).

### Cis-regulatory elements

The sequences corresponding to 1,600 bp genomic regions upstream of each GmASMTs were downloaded from the Phytozome^7^ and analysed for the presence of cis-regulatory elements using the Plant CARE.^8^

### RNA-seq and co-expression network analysis

All the experimental RNA-seq data were obtained from European Nucleotide Archive (ENA), and soybean genome, FASTA, and GTF files were obtained from Ensemble Plants. The reads were aligned using RNA STAR (Dobin et al., 2013) using the soybean genome, and aligned reads were counted using FeatureCounts (Liao et al., 2014) in the galaxy server (Afgan et al., 2018). The differential expression analysis was performed in DESeq2 (Love et al., 2014) in R (Core, 2015). The experimental data, accession number, and conditions are mentioned in Supplementary Table S1. Co-expression gene networks were performed by WGCNA to explore the highly correlated genes modules in the samples (Langfelder and Horvath, 2008). In the scale-free network, soft powers of *β* = 10 (abiotic stress) and 14 (embryo development and biotic stress) were selected using the pickSoftThreshold function (Supplementary Figure S1). The genes in the given modules had a ≥ 0.5 correlation among the genes. All the constructed network from WGCNA was visualized using Cytoscape 3.9.1. The candidate gene in the modules was annotated using Database for Annotation Visualization and Integrated Discovery (Dennis et al., 2003). The entire workflow for the RNA-seq dataset analysis is given in Supplementary Figure S2.

### Soybean seed priming

Soybean seeds, variety KHSB_2, were obtained from the University of Agricultural Sciences, Bengaluru, India. The seeds were surface sterlized for in-vitro propagation, and sterilized seeds were soaked in 10, 50, and 100 μM SER and MEL concentrations for seed priming. These seeds were kept at 25°C in the dark for 16 h under constant shaking. After 16 h, the primed seeds were washed with distilled water, followed by drying with blotting paper. Seeds were randomly selected and inoculated on MS basal medium supplemented with 3% sucrose (w/v) and 0.3% (w/v) phytagel. The culture bottles were maintained in the tissue culture room at 24°C, under a 16/8 h photoperiod, for 12–14 days to obtain germination.

### RNA isolation and qPCR

The RNA was extracted from 14-day seedlings (cotyledonary) from control and treated plants using a spectrum plant total RNA kit from Sigma-Aldrich, India. RNA was quantified using Nanodrop (ND1000, Thermo Scientific, United States). A volume of 1 μg of total RNA was reverse transcribed into cDNA using a Verso cDNA Synthesis Kit (Thermo Scientific). All the gene-specific primers (Supplementary Table S2) were designed using an oligo-analyser from Integrated DNA Technologies. The qPCR analysis was accomplished using Applied Biosystems QuantStudio™5 with 10 μl reactions containing 5 μl of SYBR Green Master Mix (TAKARA) as previously reported (Kumar et al., 2021b).

### Cloning of GmASMT and enzyme activity assay

The full-length GmASMT-specific primers were amplified by PCR. The full-length gene sequences of GmASMT33 and GmASMT44 was cloned and submitted to the NCBI (MW790258 and MW790259). The cloned genes were further amplified using a primer set containing SacI and HindIII restriction sites. The resulting amplicons were ligated into pET28a in the reading frame through the same restriction sites. Protein is overexpressed in BL21 with 0.5 mM isopropyl-b-D-thiogalactopyranoside and 2.5 mm N-Acetylserotonin at 28°C for 6 h. The supernatant of the cell lysate was subjected to HPLC.

### HPLC conditions for MEL assay

HPLC analysis was achieved using a Shimadzu model LC-20A (Shimadzu Co., Japan) coupled with a fluorescent detector, and separation was achieved using a YMC-Triart column (250 mm × 4.6 mm and 3 μm). A linear HPLC gradient composed of mobile phase A (0.4% formic acid and water) and B (0.4% formic acid and methanol) was used for the separation at a flow rate of 0.4 ml/min for 35 min. N-Acetylserotonin and MEL were detected at an excitation and emission wavelength of 280 and 350 nm, respectively. The standards and enzyme assay chromatogram is presented in Supplementary Figure S3.

### Homology modelling

The homology model of cloned GmASMT33 and GmASMT44 was built using different methods (GalaxyWEB, Modeller, and reporter X). Based on the scores from the online tools, SAVE v60,^9^ and Swiss structure assessment,^10^ we have selected the models built by GalaxyWEB^11^ for both putative GmASMTs.

### Molecular docking

For docking, the structure of N-Acetylserotonin was obtained from PubChem.^12^ The molecule is prepared using Autodock Tools,^13^ where polar hydrogen was added, and the files were saved as PDBQT. Docking simulation was conducted by Autodock Vina using the PyRx interface (Dallakyan and Olson, 2015). The resulted docking results were analysed using the molecular visualisation tools UCSF Chimera.^14^ The 2D plots for molecular interactions of surrounding molecules with the ligands were generated using BIOVIA Discovery Studio Visualizer.^15^

### Statistics

The statistical analysis for the reference genes (18 s, 60s, ELF, CY4, CDK, UBQ-E, and UBQ-F) was performed using the R packages NormqPCR. The selection of the most stable reference gene was made using geNorm (Supplementary Tables S3, S4). Relative gene expression levels were calculated using the 2^–ΔΔCT^ method (Rao et al., 2013). The significance was calculated using a two-tailed t-test with a False Discovery Rate (FDR). Differential gene expressions are represented as log2 fold change and are used in Heatmap and Volcano plots.

## Results

### The GmASMT family

The hidden Markov model identified 44 candidate ASMTs in soybean. The GmASMT names are assigned as GmASMT1 to GmASMT44 according to the soybean chromosome locations. The GmASMTs information regarding its locus name, amino acid length, MW, and pI are listed in Supplementary Table S5. The protein length of GmASMTs ranged from 219 to 378 amino acids, MW ranged from 25.2 to 41.74 kDa, and pI ranged from 4.85 to 9.07. Analysis of the conserved motifs of GmASMT proteins by employing MEME tools and CDD reveals that the GmASMT sequences contained the domains; dimerization, and S-adenosylmethionine-dependent O-methyltransferase, based on CDD. Motifs 2 and 5 encode for dimerization, whereas 1, 3, and 5, encode for the O-methyltransferase domain (Supplementary Figure S4).

### Chromosome locations and duplication

**Figure 1:**
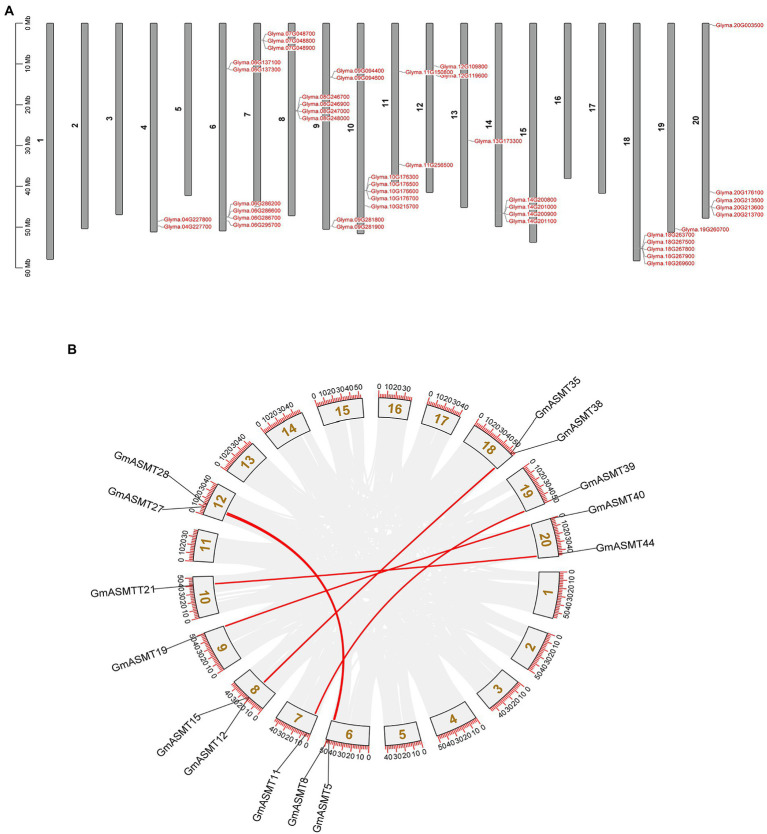
**(A)** The chromosomal location of all the 44 putative GmASMTs is distributed among 20 chromosomes. **(B)** The genome-wide chromosome organization is caused by whole-genome duplication in the Glycine max genome. Paralogous pairs of duplicated genes are grey coded, whereas GmASMTs genes are connected with red lines.

A total of 44 GmASMTs were situated among 20 chromosomes, and the genes were unevenly distributed between the chromosomes. The maximum number of six genes were present on the 6th chromosome, and five genes on the 10th, 18th, and 20th chromosomes. Most of the GmASMT were clustered at the terminal end of the chromosomes. The locations of GmASMT duplications in the soybean genome are shown in Figure 1. There are seven pairs of tandem and segmental duplications at present in soybean (Figures 1A,B). The tandem duplications are present on the 4–8th, 10th, 14th, and 20th chromosomes. Segmental duplications are present between 20–09, 19–07, 12–06, 18–08, and 20–10 chromosomes (Figure 1).

### Phylogeny analysis

**Figure 2:**
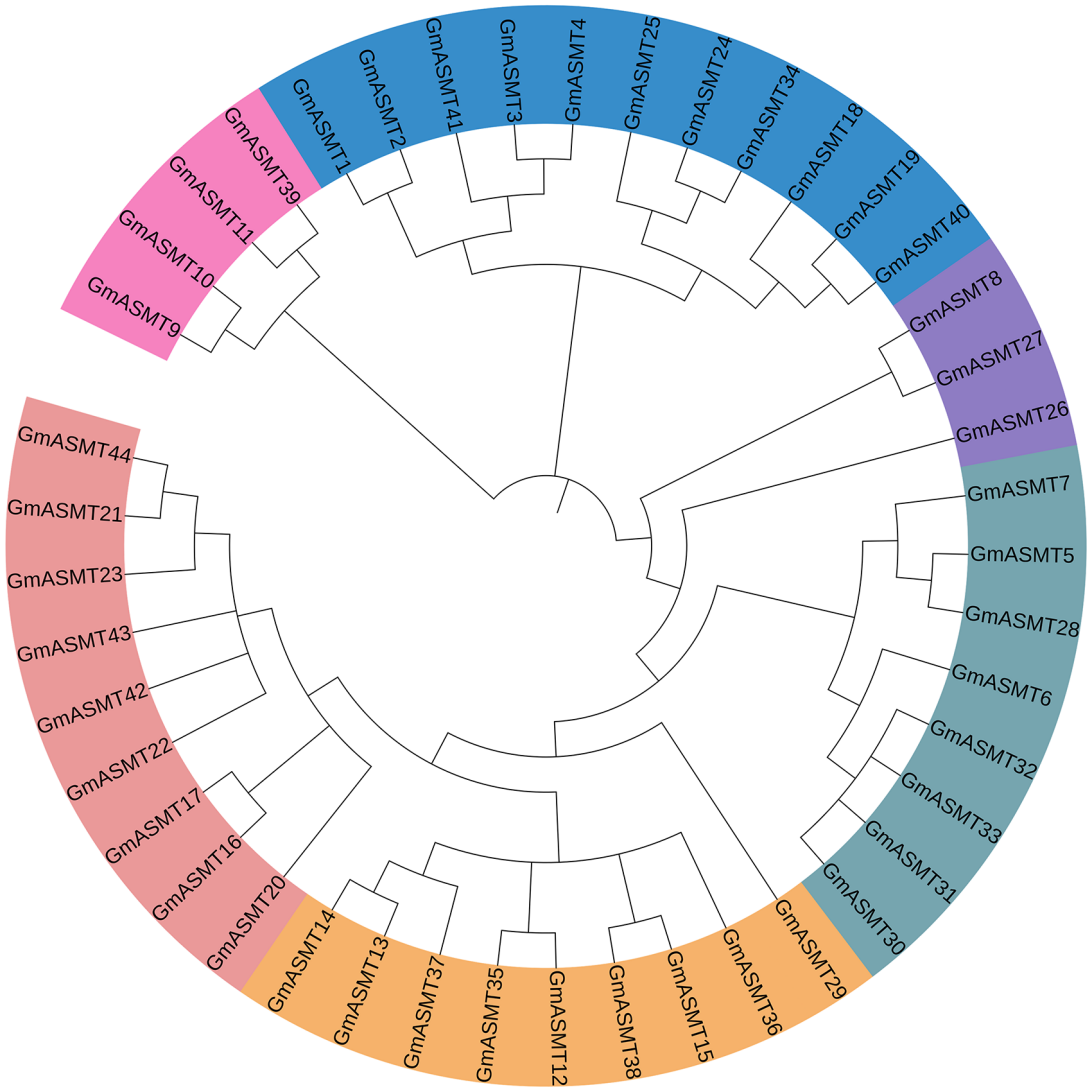
Phylogenetic analysis of all putative GmASMTs. The sequences were aligned using MUSCLE after refinement of gene annotations, phylogenetic analysis was performed using the maximum likelihood method in MEGAX. The different colours represent different GmASMT groups in Glycine max.

The Neighbour-joining phylogenetic tree was constructed among 44 GmASMT protein sequences in MEGAX, and GmASMT IDs are shown in Figure 2. Based on the phylogenetic analysis, ASMT in soybean is clustered into many branches from the root. We have grouped them into six groups and represented them with different colours (Figure 2). These results indicated significant sequence variations among different subsets of soybean ASMTs. The Ks and Ka substitution rates are used to estimate the GmASMTs duplicate divergence times. All tandem duplicates had Ks values (0.312–0.026) and segmental duplicates (0.250–0.159), indicating that they had a relatively earlier origin (Supplementary Table S6). In addition, GmASMT10 and GmASMT9 had higher Ks values and were significantly greater than those of other tandem duplicates, showing that they are derived from more ancient duplication events.

### Cis-element analysis

The analyses of GmASMTs cis-regulatory elements revealed the presence of various stress-responsive elements (temperature, defence, wound, and drought), hormone-responsive elements (auxin, abscisic acid, salicylic acid, methyl jasmonate, and gibberellin), light-responsive elements, and seed and endosperm responsive-elements in GmASMT promoters. Our results show that all the GmASMT contains multiple cis-elements in the promoter region in addition to other basic promoter elements (Supplementary Figures S5–S7).

### Expression analysis

**Figure 3:**
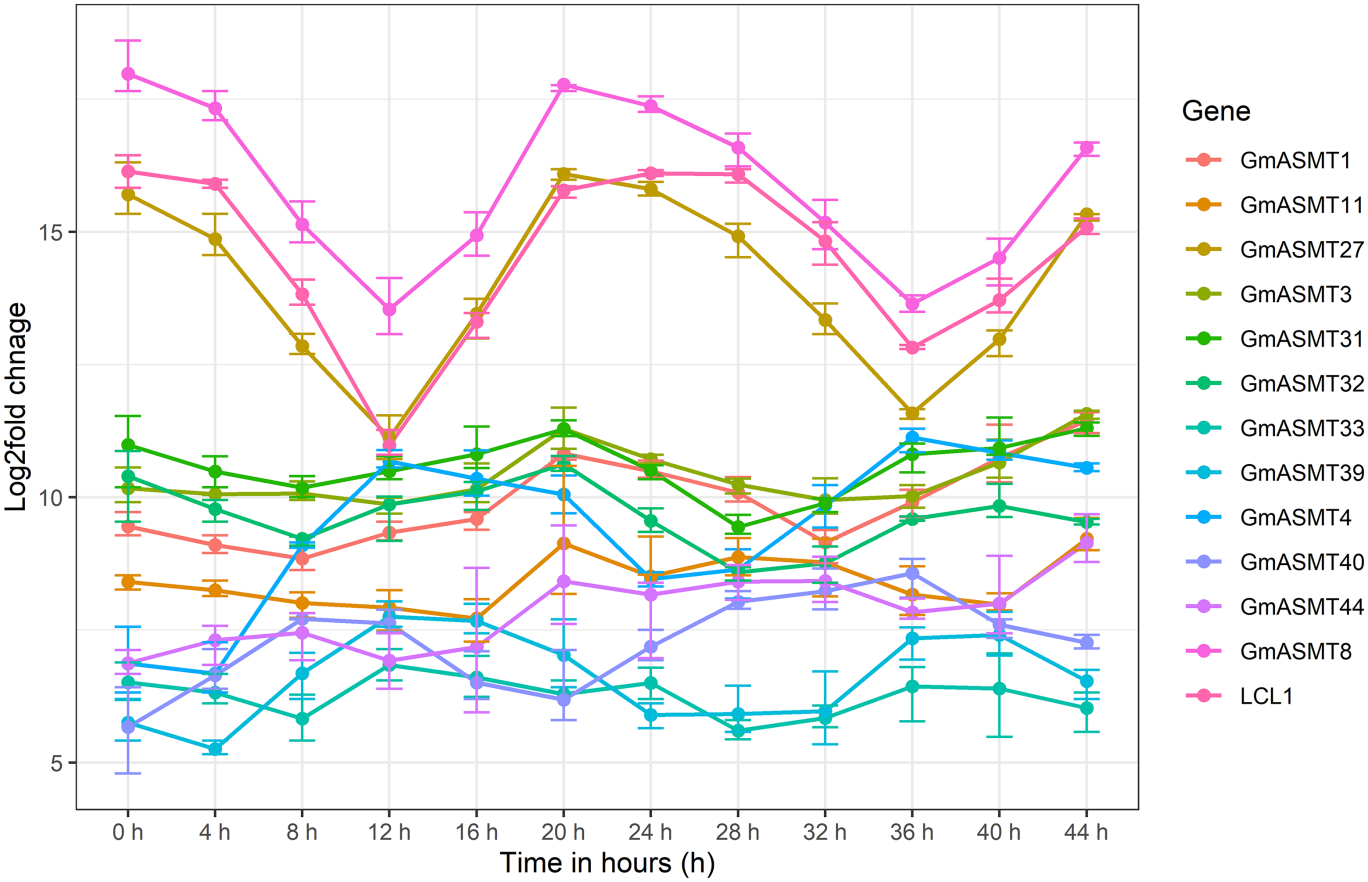
The line graph of GmASMTs and LCL1 gene expression during the time interval of 4 h for the duration of 44 h. The different colour line indicates the GmASMTs as indicated in the Figure key on the right side, *x*-axis represent the time in hours, *y*-axis represent the log2fols change of genes.

The expression profile of 12 GmASMTs was detected in the circadian rhythm RNA-seq data along with LCL1 as a circadian control gene. Among 12 GmASMTs, GmASMT27, and LCL1 had demonstrated identical peak expression after 0, 24, and 44 h, and higher down-regulation at 12 and 36 h shows the probable involvement during the circadian cycle (Figure 3).

**Figure 4:**
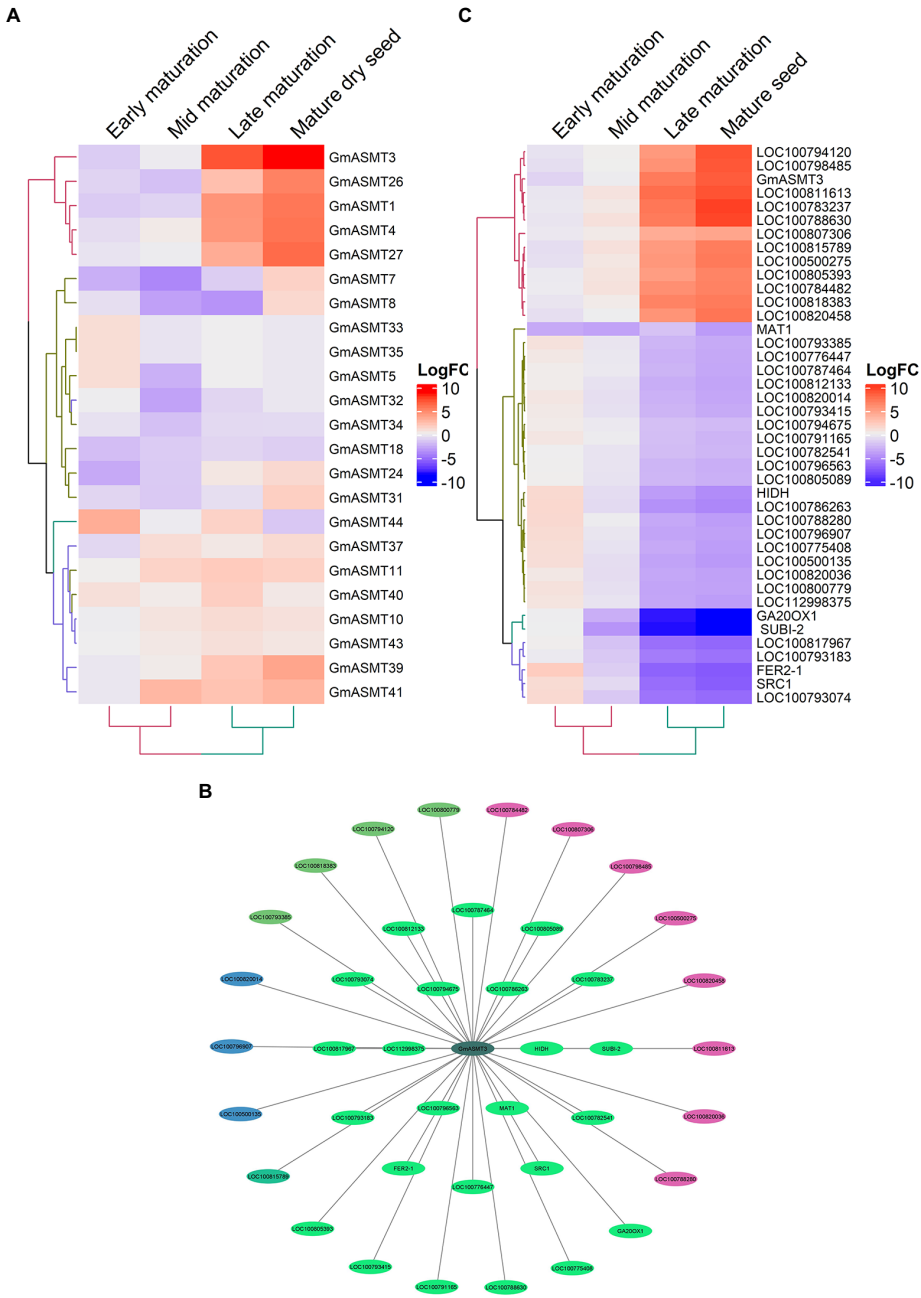
**(A)** The Heatmap of GmASMTs expression under embryo developmental stages. **(B)** Network genes of GmASMT3 in centre and all the interacting genes are represented around the GmASMT3, the different colours of nodes represent the different genes clusters and the arrow indicates the direction. **(C)** The expression of all the network genes associated with GmASMT3 under different stages of embryo development.

After analysing RNA-seq data of cotyledonary embryo, early maturation, mid maturation, late maturation, and mature-dry seeds for GmASMTs expression, we observed that many GmASMTs showed significant upregulation during late maturation and dry seed stages, compared to early and mid-maturation stages (Figure 4A; Supplementary Figure S8A). The WGCNA revealed GmASMT3 interacting with 41 network genes present in different gene modules, which are represented in a different colours (Figure 4B). Functional analysis of network genes of GmASMT3 by DAVID revealed the genes are related to secondary metabolites, photosynthesis, glycolysis, MAPK signalling pathway, plant hormone signal transduction, and protein processing in the endoplasmic reticulum. GmASMT3 also showed interaction with 2-hydroxyisoflavanone dehydratase (HIIDH) involved in isoflavone biosynthesis (daidzein). The list of 41 genes names and functions is given in Supplementary Table S7. The gene expression of 41 network genes under different development stages is given in Figure 4C.

**Figure 5:**
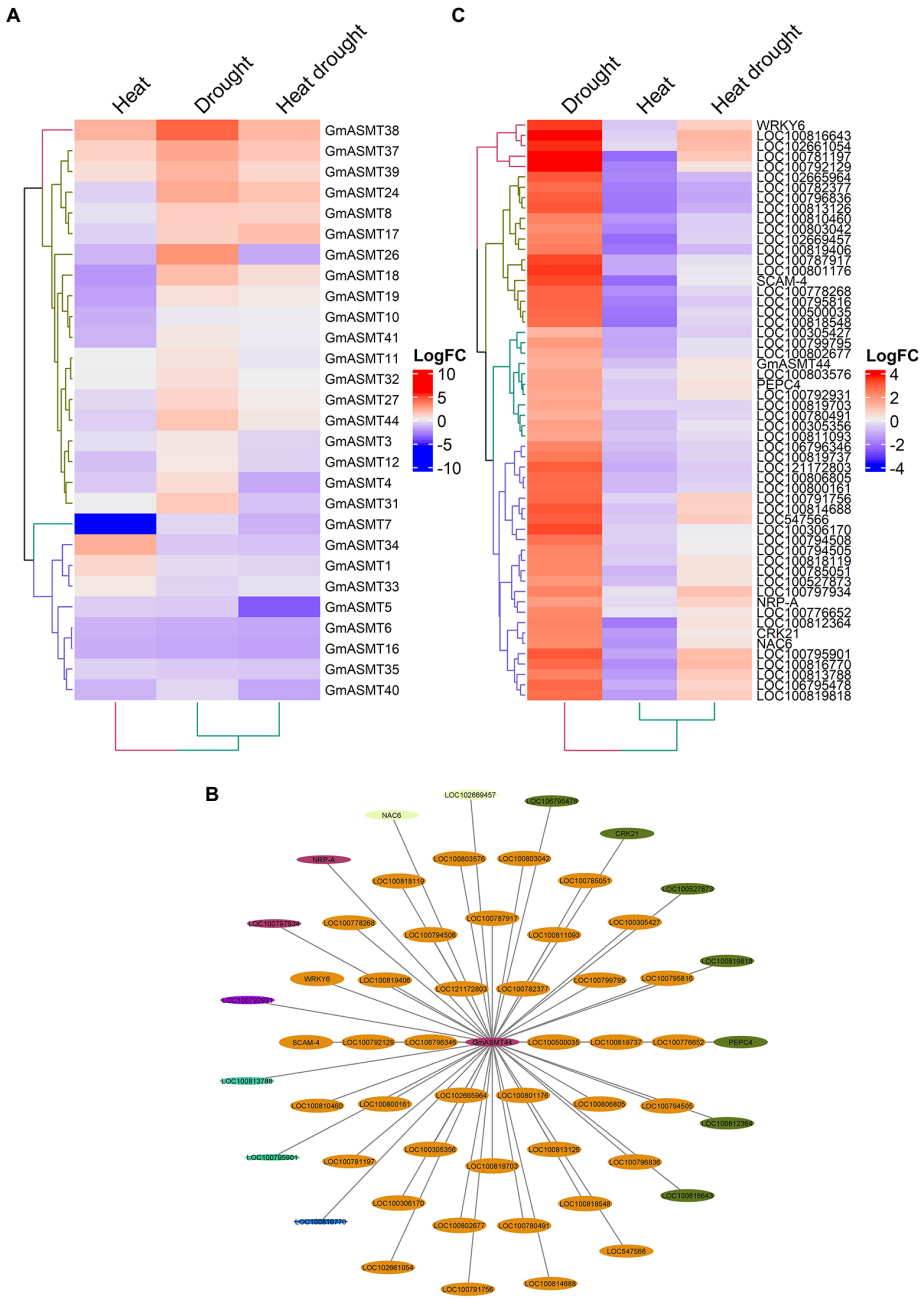
**(A)** The Heatmap of GmASMTs expression under heat and drought stress. **(B)** Network genes of GmASMT44 in centre and all the interacting genes are represented around the GmASMT44, the different colours of nodes represent the different genes clusters and the arrow indicates the direction. **(C)** The expression of all the network genes associated with GmASMT44 under heat and drought stress.

To determine the importance of GmASMTs under abiotic (heat, drought, and combined heat and drought) stress, we detected 28 GmASMTs from the RNA-seq data (Figure 5A). The significant up and downregulation of GmASMTs under abiotic stress are given in Supplementary Figure S8B. Among all the detected GmASMTs, WGCNA revealed GmSNAT44 as interacting with the 55 networks from genes from six different modules represented with different colours (Figure 5B). The expression of 55 network genes of GmASMT44 is given in Figure 5C. The GmASMT44 is associated with stress response, transcription regulation, hormone signalling, calcium ion binding, protein kinase activity, and carbon metabolism (Supplementary Table S8). Among 55 genes associated with GmASMT44, 52 genes were annotated using DAVID, and the list is given in Supplementary Table S8. Under abiotic stress, GmASMT44 is involved in regulating the expression WRKY6 and NAC6 transcription factors involved in stress regulation in plants.

**Figure 6:**
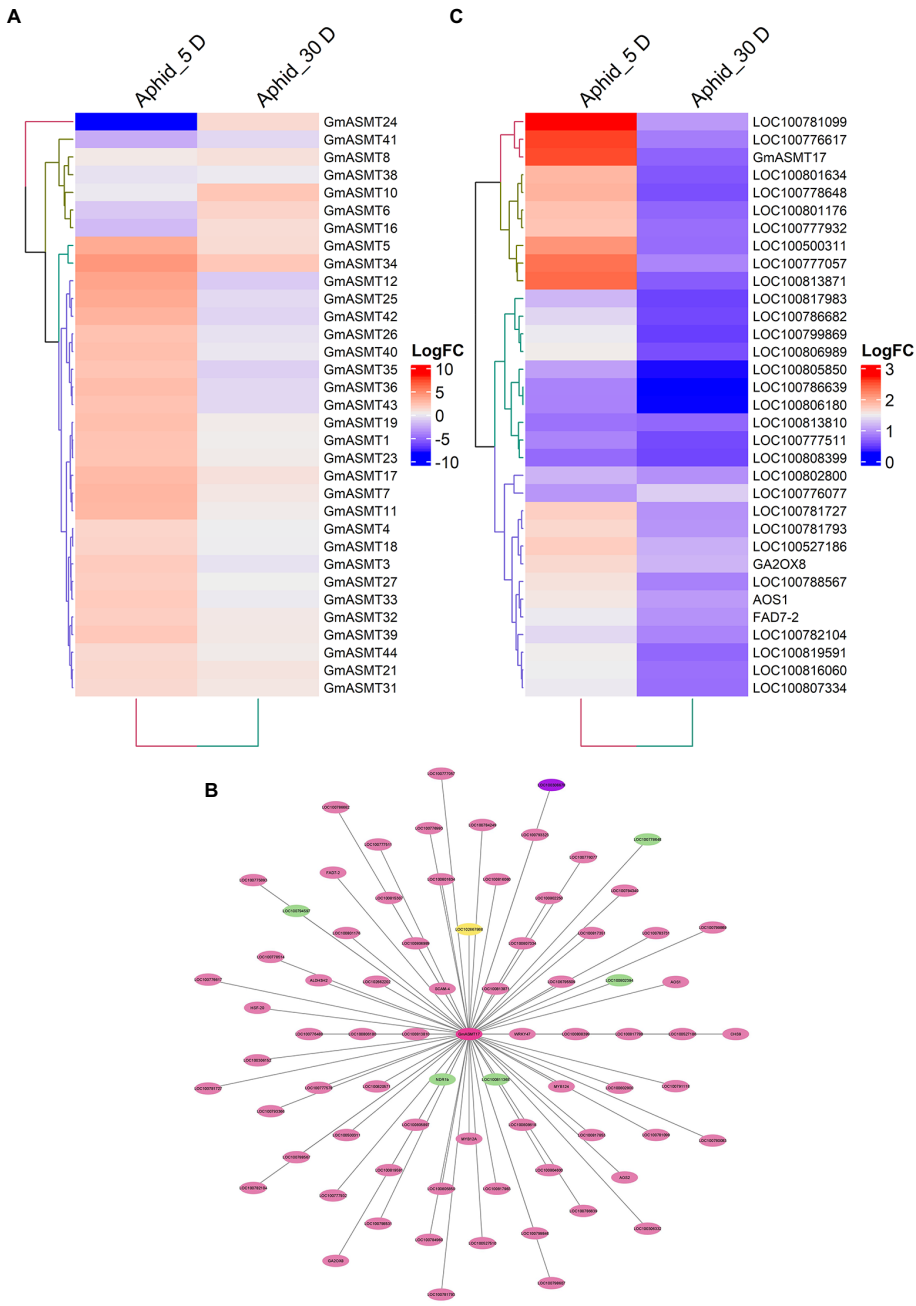
**(A)** The Heatmap of GmASMTs expression under aphid infections. **(B)** Network genes of GmASMT17 in centre and all the interacting genes are represented around the GmASMT17, the different colours of nodes represent the different genes clusters and the arrow indicates the direction. **(C)** The expression of all the network genes associated with GmASMT17 under aphid infections.

The aphid infections have greatly influenced the expression of 33 GmASMTs detected in the RNA-seq data (Figure 6A). The significant up and downregulation of GmASMTs under aphid infections are given in Supplementary Figure S8C. The WGCNA of RNA-seq data revealed that GmASMT17 interacted with 76 network genes associated with three different clusters (Figure 6B). The GmASMT17 network genes interacted with transcription factors (MYB, WRKY, NAC, and ERF), calmodulin protein, plant hormone signal transduction, stress signal, hydrolase, isoflavone biosynthesis, jasmonic acid biosynthesis, and phosphatase activities (Supplementary Table S9). The expression of network genes associated with GmASMT17 under aphid infections is given in Figure 6C.

**Figure 7:**
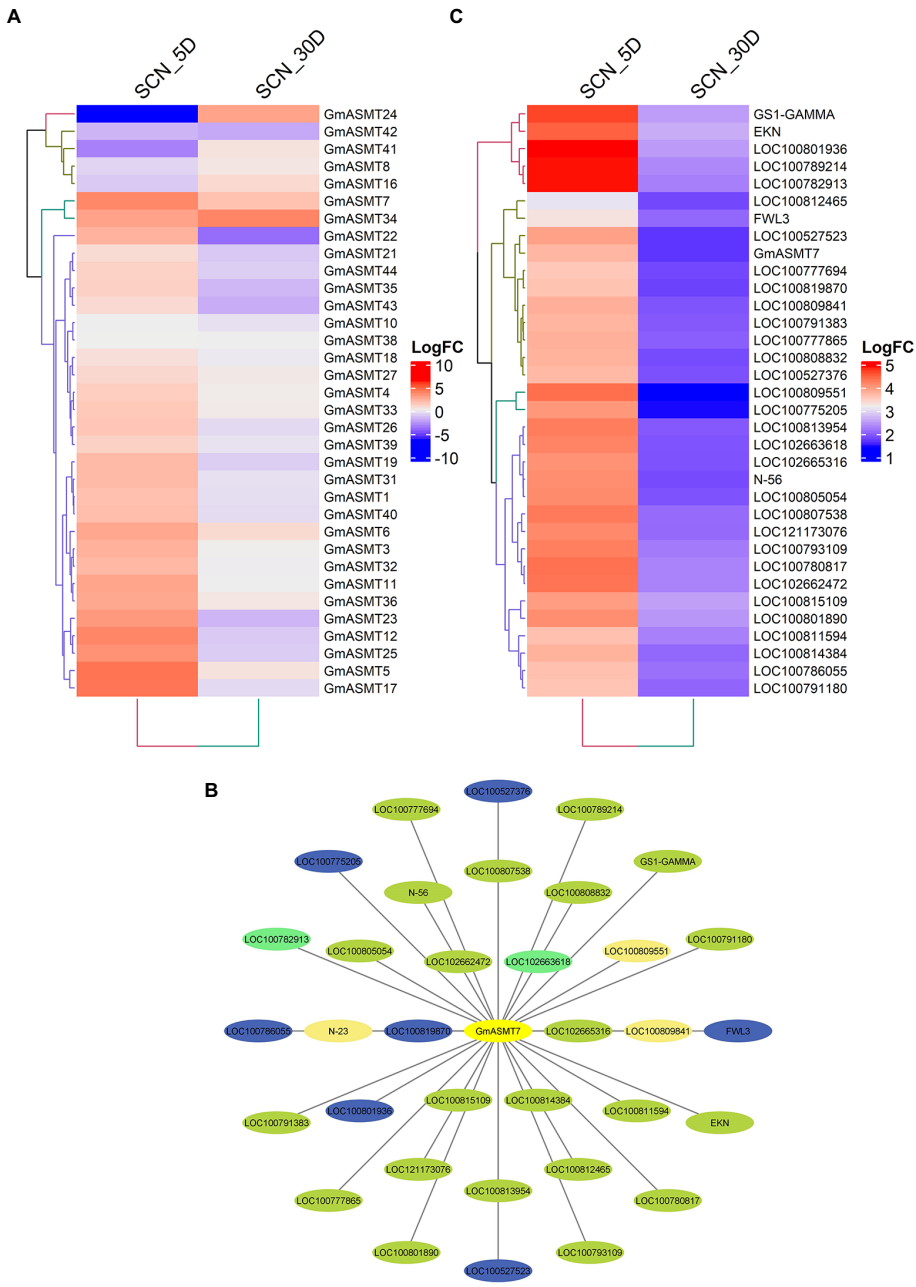
**(A)** The Heatmap of GmASMTs expression under soybean cyst nematode (SCN) infections. **(B)** Network genes of GmASMT3 in centre and all the interacting genes are represented around the GmASMT3, the different colours of nodes represent the different genes clusters and the arrow indicates the direction. **(C)** The expression of all the network genes associated with GmASMT3 under soybean cyst nematode (SCN) infections.

We detected 34 GmASMTs in SCN infection RNA-seq data, and all the GmASMTs showed varied expression at 5 and 30 days intervals (Figure 7A). The significant up and downregulation of GmASMTs under SCN infections are given in Supplementary Figure S8D. The GmASMT7 has interacted with 34 network genes belonging to four different clusters during SCN infections (Figure 7B). The network genes are associated with numerous membrane transporter, amino acid biosynthesis, a signalling protein, ubiquitin, defence responsive, protein phosphorylation, and cell-wall biosynthesis (Supplementary Table S10). In addition, the expression of the 34 network genes of GmASMT7 under SCN infection during 5 and 30 days is given in Figure 7C.

### Expression analysis of GmASMTs under SER and MEL seed priming

**Figure 8:**
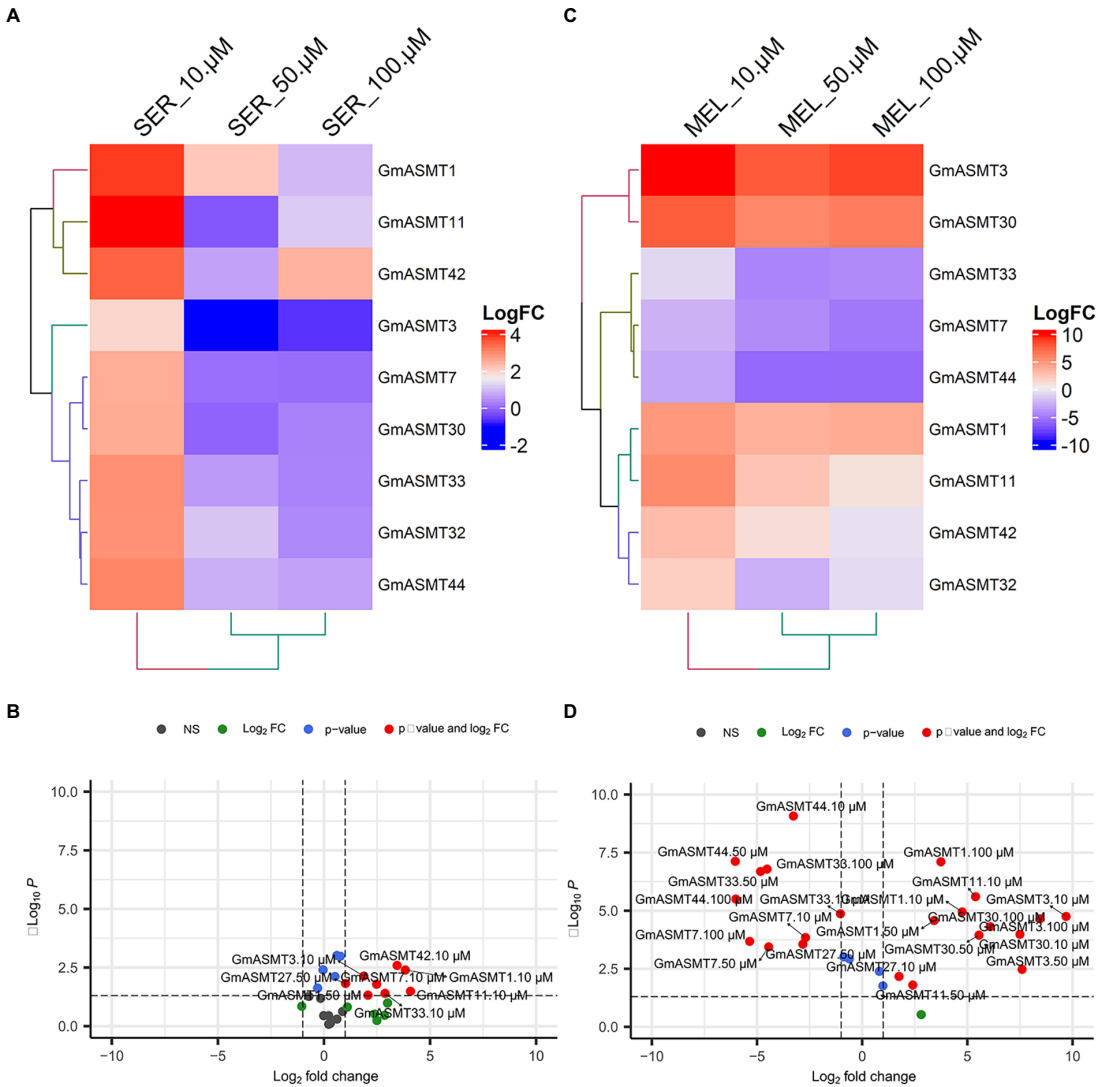
**(A)** Heatmap and **(B)** volcano plots showing GmASMTs expression under different concentrations of SER seed priming. **(C)** Heatmap and **(D)** volcano plots showing GmASMTs expression under different concentrations of MEL seed priming.

To understand the effect of different concentrations of SER and MEL on the expression of GmASMTs, we quantified the expression of GmASMT1, GmASMT3, GmASMT7, GmASMT11, GmASMT30, GmASMT32, GmASMT33, GmASMT40, and GmASMT44. In the presence of 10 μM concentration of SER, all the GmASMTs have shown the maximum upregulation compared to 50 and 100 μM SER concentrations (Figures 8A,B). However, MEL treatment significantly upregulated the expression of GmASMT3, GmASMT30, GmASMT1, and GmASMT11 in all the concentrations (Figures 8C,D).

### Cloning of full-length GmASMT

GmASMT44 and GmASMT33 full-length putative GmASMT were cloned from the cotyledonary leaves of *G. max*. The Sanger sequencing analysis of cloned putative GmASMT, GmASMT33, and GmASMT44 revealed an open reading frame (ORF) of 1,077 and 1,065 bp, respectively, which encoded a predicted protein of 358 and 354 amino acids with a calculated molecular weight of 39.80 and 40.42 kDa and theoretical pI value of 5.45 and 5.83, respectively. These two-share similarities of 43.50 with each other and with *A. thaliana* and *O. sativa* around 36–40%.

### Homology modelling

The final predicted models of GmASMT, GmASMT44, and GmASMT33 were generated by homology modelling and visualized using chimera (Supplementary Figures S9A,B). The PDB files with the greatest identity with the query sequences were selected. The structure was verified using Verify3D, where it showed an overall quality score of 96.158 and 96.788 for GmASMT44 and GmASMT33. Additionally, GmASMT44 and GmASMT33 showed the highest number of residues, 97.16 and 95.79%, within the preferred regions of the Ramachandran plot and the least number of outliers. Therefore, the structures can be used for further molecular docking and simulation studies.

### Molecular docking

ASMT is a homodimer protein that contains a pair of identical subunits, but in the current study, only one subunit was predicted for structure predictions (Supplementary Figure S9). After performing the blind dock between putative GmASMT33 and GmASMT44 and N-Acetylserotonin, we found a binding affinity of −6.2 and − 6.4 Kcal/mol was observed. The 2D view of the protein-ligand complex of the best poses generated by the auto dock is shown in Supplementary Figures S9C,D. GmASMT33 interactions can be found between N-Acetylserotonin atoms and the GmASMT33 amino acid residues Phe118, Phe178, Leu350, and Arg351 (Supplementary Figure S9D). Between N-Acetylserotonin and GmASMT44 amino acid residues Phe120, Arg125, Ala152, Trp153, Phe166, Asn260, and Met316 (Supplementary Figure S9E). These amino acids directly participate in the catalytic mechanism of this ASMT. The hydrophobicity around the ligand-enzyme complex of both GmASMTs is shown in Supplementary Figures S9C,D. The top-ranked binding pose with the least docked binding affinities, and high docking scores are used as a standard selection of the docking programs. The best poses of GmASMT33 and GmASMT44 with N-Acetylserotonin generated by Auto Dock. The binding affinity is found to be −6.2 and − 6.4 Kcal/mol. The various interactions of van der Waals, conventional hydrogen bonds, Pi-Donor hydrogen bonds, Pi-alkyl, Pi-Pi T-shaped, carbon-hydrogen bonds, and Pi-sulfur are observed between putative GmASMT 33 and GmASMT44 with N-Acetylserotonin (Supplementary Figures S9D,E).

## Discussion

In our recent studies (Kumar, et al., 2021a,b), we have noticed the importance of MEL in growth, temperature stress tolerance, and increased isoflavones contents in soybean. ASMT is the last enzyme involved in the MEL biosynthesis pathway catalysing the methyl group to acetyl serotonin and forming MEL. Studies on the expression of ASMT will contribute in understanding the regulatory and biosynthesis of MEL in plants (Ma et al., 2022). However, it is still not being screened or identified in soybean. Therefore, the present study was focused on genome-wide identification of ASMT in soybean through HMM, where we observed 44 putative ASMT genes in soybean. Whereas, in *Solanum lycopersicum* (Liu et al., 2017) and *Capsicum annuum* (Pan et al., 2019), 14 and 16 ASMT genes were found. The greater number of putative GmASMTs in the present study is due to the size and duplication event of the soybean genome, and a similar observation was seen in lectin receptor-like kinases (Liu et al., 2018) and BES1 transcription factor family in soybean (Li et al., 2019).

The presence of many genes indicates a vital role in the course of plant evolution (Liu et al., 2017). The gene duplication events are one of the primary driving forces in the evolution of gene families with variations in soybean (Zhao et al., 2020). Consequently, we found seven pairs of segmental and tandem-duplicated GmASMTs (Figure 1) were found in soybean, and duplication events tend to have different fates in transcriptional evolution by cis- and trans-regulatory divergence in soybean (Zhao et al., 2020). Our analysis of cis-regulatory elements (Supplementary Figures S5–S7) revealed the presence of diverse regulatory elements involved in the development, stress response, and circadian rhythm. The expression pattern and cis-regulatory region of duplicated genes were highly variable among the GmASMTs. These observations suggest the pleiotropic nature of GmASMTs, making it one of the vital genes involved in various physiological and biochemical functions in soybean.

Numerous expression datasets (Supplementary Table S1) enabled us to investigate the expression of genes and quantify their abundances across treatments, thus revealing the crucial role of genes. By utilizing circadian rhythm datasets, we found that GmASMT27 showed circadian clock oscillation (Figure 3), a characteristics feature of LCL1, involved in maintaining circadian rhythmicity in plants (Wang et al., 2020). Recent reports also demonstrated the importance of circadian rhythm in various physiological processes, metabolic pathways, developmental and degenerative processes (Venkat and Muneer, 2022).

Co-relation networks across conditions revealed the potential regulatory mechanism of GmASMTs. For example, in embryo developmental stages, we identified the key role of GmASMT3, clustered with 41 genes involved in various biological activities (Figure 4). Among them, 2-hydroxyisoflavone dehydratase, a limiting factor in the (daidzein) isoflavone biosynthesis expression (Malla et al., 2020), was upregulated at early and mid-embryo maturation developments, and this enzyme is present only in plants producing isoflavones (Gupta et al., 2017). Accordingly, isoflavone content gradually increases from the early to late embryo development stages of soybean (Devi et al., 2020), and MEL treatment is also known to increase the isoflavones content (Kumar et al., 2021b). Another member of the GmASMT3 network of genes, transporters: ABC transporter and WAT1, where ABC transporter transport complex building blocks essential for the plant cell development during embryo development (Do et al., 2018), and we observed upregulation of early embryo development stages (Figure 4; Supplementary Table S7). Whereas WAT1, involved in the plant hormone auxin transport, shows upregulation during the late stages of embryo development (Figure 4A) and plays a crucial role in integrating auxin signalling, cell-wall formation, and development of plants (Ranocha et al., 2013; Casanova-Sáez and Voß, 2019; Lv et al., 2021).

After deciphering the importance of GmASMT during embryo development, we extended our studies to understand abiotic and biotic stress in soybean. The co-relation network revealed the GmASMT44 network of 55 genes under heat and drought stress. Among 55 genes of GmASMT44, three of them were related to lectin S-receptor which plays vital roles in sensing exterior abiotic and biotic stress signal perception and transduction in plants (Sun et al., 2013, 2020). Additionally, GmASMT44 also showed interactions with NAC and WRKY transaction factors, which regulate stress tolerance gene expression in response to abiotic and biotic stresses (Erpen et al., 2018; Javed et al., 2020). Previously ASMT is reported to recruit WRKY to regulate MEL biosynthesis in cassava (Wei et al., 2018). In abiotic stresses, IAA is involved in the regulatory networks with antioxidant enzymes and calcium-binding protein, which are strongly affected by environmental stresses (Tognetti et al., 2012; Singh et al., 2021; Zhang et al., 2021). Similarly, we observed that the network genes of GmASMT44 are associated with auxin-responsive protein, calcium, and calmodulin-binding protein involved in the regulatory mechanism of stress responses (Figure 5; Supplementary Table S8).

In order to understand biotic stress, we analysed aphid and SCN infections of soybean plants. Under aphid infection, through networking, we identified GmASMT17, which showed interactions with 42 genes associated with different stress responses (Figure 6). GmASMT17 interacts with transcription factors like MYB, ERF, NAC, and WRKY, which has the potential to regulate abiotic and biotic stress-related genes in plants (Erpen et al., 2018). Further, GmASMT17 showed interactions with allene oxide synthase involved in jasmonic acid biosynthesis, where soybean is known to induce the expression of phytohormones biosynthesis pathways like abscisic acid and jasmonic acid (Chapman et al., 2018). In SCN infectious condition, we identified that GmASMT7 interacted with 34 genes involved in various regulatory mechanisms of the biotic stress response (Figure 7). Where GmASMT7 showed interaction with membrane proteins like CBS domain-containing protein, F-box protein, and RING-H2 finger protein, which are known to play significant roles in abiotic stress responses in plants (Zhou et al., 2015; Song et al., 2016; Kumar et al., 2018). GmASMT7 also interacts with three NRT1/PTR family proteins which act as plant hormone transporters like auxin, abscisic acid, and secondary metabolites (Chiba et al., 2015). Similarly, in transcriptome analysis of Arabidopsis after MEL and auxin treatments, most of the auxin-regulated genes were co-regulated with MEL, indicating that MEL and auxin were regulated by a similar subset of genes (Yang et al., 2021).

In this study, GmASMT33 and GmASMT44 were cloned and functionally evaluated, where GmASMT44 showed its importance in response to heat and drought stress (Supplementary Figure S6). These two GmASMTs are verified in the bacterial system, where both were able to convert N-Acetylserotonin to MEL, showing ASMT enzyme activity (Kang et al., 2011; Byeon et al., 2016). The crystal structure of Homo sapiens ASMT is reported to share a resemblance with the chalcone O-methyltransferase and isoflavone O-methyltransferase (Botros et al., 2013). Similarly, GmASMT33 shared 46.45 and 46.95, and GmASMT44 shared 57.51 and 55.77, respectively. Therefore, these crystal structures were selected for homology modelling to identify the active amino acid interactions with the ligand of cloned GmASMTs (Supplementary Figures S9A,B).

Various studies have reported that MEL seed priming leads to higher rates of photosynthesis, transpiration, and gas exchange in plants (Cao et al., 2019; Sorrentino et al., 2021). In comparison, SER and MEL seed priming in plants and its role in seed germination and expression of GmASMTs has not been studied so far. Therefore, SER and MEL priming was taken up to understand the mechanism of soybean seed germination and its effect on the putative GmASMT involved in MEL biosynthesis. This can be further studied by challenging the plants to perform against abiotic and biotic stress in soybean. The presence of 10 μM SER concentration seed priming led to the highest increase in seedling growth and expression of all the screened GmASMTs. Similarly, in soybean cell cultures, the SER has significantly increased cell biomass and isoflavones content (Kumar et al., 2021a). These results show that the presence of SER increased the expression of GmASMTs involved biosynthesis of MEL in soybean. The enzyme assay of GmASMT33 and GmASMT44 with N-acetyl serotonin showed the formation of MEL product which is quantified using HPLC.

## Conclusion

The pleiotropic nature of MEL has to be associated with the ASMT involved in MEL biosynthesis. Multiple GmASMTs are due to the duplication event in the soybean genome. Analysis of the GmASMTs promoter region revealed light, circadian, drought, cold, and phytohormones responsive elements, which play a vital role in the production of MEL by initiating GmASMT expression, and the same is confirmed with expression analysis, where distinct GmASMTs are involved in developmental, biotic and abiotic stress. In addition to expression studies, network analysis proved that GmASMT would interact with the auxin receptor/transporter/regulator in plants, and that’s why many studies are reporting that MEL shows auxin-like activities in plants. Under abiotic and biotic stress, GmASMTs showed interactions with WRKY and MYB transcription factors in plant stress regulation. From all these observations, we speculate that the GmASMT plays a crucial role in the stress response in soybean. Hence GmASMT could be the primary target in genetic engineering to achieve crop improvement under changing environmental conditions.

## Data availability statement

The datasets presented in this study can be found in online repositories. The names of the repository/repositories and accession number(s) can be found in the article/Supplementary Figure S6.

## Author contributions

The experiment was supervised by PG and BP, who also assisted in editing the manuscript. GK, RP, and MA performed the experiments and assisted in preparing the manuscript. GK designed, conducted, and wrote the manuscript. All authors participated in manuscript preparation and review. All authors contributed to the article and approved the submitted version.

## Funding

GK, RP, and MA are grateful to ICMR, New Delhi, DST, New Delhi, and DBT, New Delhi, respectively, for the fellowships.

## Conflict of interest

The authors declare that the research was conducted in the absence of any commercial or financial relationships that could be construed as a potential conflict of interest.

## Publisher’s Note

All claims expressed in this article are solely those of the authors and do not necessarily represent those of their affiliated organizations, or those of the publisher, the editors and the reviewers. Any product that may be evaluated in this article, or claim that may be made by its manufacturer, is not guaranteed or endorsed by the publisher.

## Footnotes

^1^https://www.ebi.ac.uk/Tools/pfa/pfamscan/ (Accessed July 23, 2022).

^2^http://smart.embl-heidelberg.de/ (Accessed July 23, 2022).

^3^https://web.expasy.org/compute_pi/ (Accessed July 23, 2022).

^4^https://www.ncbi.nlm.nih.gov/Structure/cdd/cdd.shtml (Accessed July 23, 2022).

^5^http://plants.ensembl.org/Glycine_max/Info/Index (Accessed July 23, 2022).

^6^http://services.cbu.uib.no/tools/kaks (Accessed July 23, 2022).

^7^https://phytozome.jgi.doe.gov/pz/portal.html (Accessed July 23, 2022).

^8^http://bioinformatics.psb.ugent.be/webtools/plantcare/html/ (Accessed July 23, 2022).

^9^https://saves.mbi.ucla.edu/ (Accessed July 23, 2022).

^10^https://saves.mbi.ucla.edu/ (Accessed July 23, 2022).

^11^http://galaxy.seoklab.org/index.html (Accessed July 23, 2022).

^12^https://pubchem.ncbi.nlm.nih.gov/ (Accessed July 23, 2022).

^13^http://autodock.scripps.edu/resources/adt (Accessed July 23, 2022).

^14^https://www.cgl.ucsf.edu/chimera/ (Accessed July 23, 2022).

^15^https://discover.3ds.com/discovery-studio-visualizer-download (Accessed July 23, 2022).
