# Supplementary material for: Distinct GmASMTs are involved in regulating transcription factors and signalling cross-talk across embryo development, biotic, and abiotic stress in soybean
Source: Front Plant Sci. 2022 Aug 11;13:948901. doi: 10.3389/fpls.2022.948901 (PMC9403468; doi:10.3389/fpls.2022.948901)
Supplement: Supplementary file 1 [file Data_Sheet_1.docx]

**Table S1.** The list of RNA-seq datasets used in the studies.

| **Stress** | **Conditions** | **Accession ID** | **No of samples** | **Experimental conditions** | **References** |
| --- | --- | --- | --- | --- | --- |
|  | Circadian cycle | GSE94228 | 48 | plants were grown in growth chamber at 28 °C under 16 h light/8 h dark photoperiod for 9 days. On the tenth day, the light was switched on to constant conditions for 24 h. The unifoliate leaves were collected on the 11^th^ day for every 4 h for 44 h duration | (Li et al., 2019) |
|  | Seed development | GSE99571 | 15 | Seed embryos were collected at four different seed developmental stages (cotyledon, early, mid and late maturation), and mature dry seeds. | (Jones & Vodkin, 2013) |
| Abiotic | Heat and drought stress | GSE153951 | 12 | The drought stress plants were supplied with 30% less water available for transpiration compared to control. The day/night temperature of control and drought groups were kept at 28/24 °C for 14/10 h photoperiod respectively. In case of heat and combined stress, the temperatures was increased from 28 to 38 °C in 8 h ramp, and then back to 28 °C on 4 h ramp and finally at 24 °C during the night as maintained in control groups | (Cohen et al., 2021) |
| Biotic | Aphid and soybean cyst nematode infections | GSE125103 | 18 | Plants were grown in a greenhouse, for SCN infection, the plants were infected with 2000 nematode eggs. For soybean aphid infection, the plants were infected with 15 soybean aphids. The roots were sampled at 5 D and 30 D post soybean aphid and SCN infestation | (Neupane et al., 2019) |

**Table S2.** List of primers used for qPCR analysis.

| **Primer name** | **Sequence (5'-3')**  **Forward primer** | **Sequence (5'-3')**  **Reverse primer** | **Accession ID** |
| --- | --- | --- | --- |
| GmASMT1 | GTGGGAAAGAGAGAACAGAGAA | CGTAGGTATTGAAAGCACAGC | Glyma.04g227700 |
| GmASMT3 | GAGGAAAAGAGAGAACCCAA | ATAACCCAGAAGCCAGAGAC | Glyma.06g131700 |
| GmASMT7 | CAACCCAAAGTGAAGAAAATGC | GTGAAGCAATGAGTTGTGAGAG | Glyma.06g286700 |
| GmASMT11 | GAGGAAAAGAGAGAACCCAA | ATAACCCAGAAGCCAGAGAC | Glyma.07g048900 |
| GmASMT30 | CATAACGAGAAAGGCAAAGA | AACCAGCAGAGGAAATCAAC | Glyma.14g200800 |
| GmASMT32 | AAATGTGTGGTTGACTTAGGCC | ATGGTTTTGTTGAGAGAAGAAG | Glyma.14g201000 |
| GmASMT33 | TGACACTACACCATTTGAAACG | AATGACTCCAATCCCATGAACAT | Glyma.14g201100 |
| GmASMT40 | GCTCTTTGGGTCCTCTCTCA | TGGAAATGGTCGTTGTTGTTAG | Glyma.20g003500 |
| GmASMT42 | GTCTCTTCAATGAGGCTATGGC | TTCCACCACCAACATCCAC | Glyma.20g213500 |
|  |  |  |  |

**Table S3.** Ranking of reference genes by geNorm method under SER treatments.

| **Rank** | **Control** | **10 µM** | **50 µM** | **100 µM** |
| --- | --- | --- | --- | --- |
| 1 | 18S | GPD | 18S | 18S |
| 1 | GPD | UBQ | GPD | ACT |
| 2 | 60S | 18S | UBQ | 60S |
| 3 | ACT | ACT | ACT | GPD |
| 4 | UBQ | 60S | 60S | UBQ |
| **M-value** | 0.00574764 | 0.01186742 | 0.006199087 | 0.013091068 |

**Table S4.** Ranking of reference genes by geNorm method under MEL treatments.

| **Rank** | **Control** | **10 µM** | **50 µM** | **100 µM** |
| --- | --- | --- | --- | --- |
| 1 | ACT | ACT | 18S | 60S |
| 1 | GPD | UBQ | UBQ | ACT |
| 2 | UBQ | GPD | ACT | UBQ |
| 3 | 60S | 18S | 60S | 18S |
| 4 | 18S | 60S | GPD | GPD |
| **M-value** | 0.005555 | 0.00664 | 0.001305 | 0.0047524 |

**Table S5.** The list of N-Acetylserotonin methyltransferase (ASMT) candidate genes in *Glycine max.*

| **Gene name** | **Gene**  **ID** | **Protein length in amino acids** | **Molecular Weight (Da)** | **Theoretical Isoelectric Point** |
| --- | --- | --- | --- | --- |
| GmASMT1 | Glyma.04G227700 | 365 | 39969.34 | 5.74 |
| GmASMT2 | Glyma.04G227800 | 333 | 36308.89 | 5.36 |
| GmASMT3 | Glyma.06G137100 | 365 | 39895.21 | 5.58 |
| GmASMT4 | Glyma.06G137300 | 365 | 39826.15 | 5.40 |
| GmASMT5 | Glyma.06G286200 | 359 | 40630.73 | 5.28 |
| GmASMT6 | Glyma.06G286600 | 352 | 39784.07 | 5.24 |
| GmASMT7 | Glyma.06G286700 | 355 | 40578.97 | 5.66 |
| GmASMT8 | Glyma.06G295700 | 369 | 41508.95 | 5.93 |
| GmASMT9 | Glyma.07G048700 | 324 | 36182.88 | 4.92 |
| GmASMT10 | Glyma.07G048800 | 354 | 38928.07 | 5.80 |
| GmASMT11 | Glyma.07G048900 | 372 | 41308.53 | 5.17 |
| GmASMT12 | Glyma.08G246700 | 322 | 36415.94 | 4.85 |
| GmASMT13 | Glyma.08G246900 | 219 | 25273.70 | 9.07 |
| GmASMT14 | Glyma.08G247000 | 284 | 32183.66 | 6.15 |
| GmASMT15 | Glyma.08G248000 | 354 | 40025.22 | 5.48 |
| GmASMT16 | Glyma.09G094400 | 353 | 40053.38 | 5.74 |
| GmASMT17 | Glyma.09G094600 | 313 | 35508.28 | 5.69 |
| GmASMT18 | Glyma.09G281800 | 369 | 41024.91 | 5.96 |
| GmASMT19 | Glyma.09G281900 | 357 | 39877.68 | 5.11 |
| GmASMT20 | Glyma.10G176300 | 273 | 30769.80 | 6.20 |
| GmASMT21 | Glyma.10G176500 | 354 | 39912.02 | 5.46 |
| GmASMT22 | Glyma.10G176600 | 355 | 39962.10 | 5.09 |
| GmASMT23 | Glyma.10G176700 | 354 | 39825.95 | 5.45 |
| GmASMT24 | Glyma.10G215700 | 369 | 40823.08 | 5.66 |
| GmASMT25 | Glyma.11G150800 | 366 | 40591.75 | 4.87 |
| GmASMT26 | Glyma.11G256500 | 366 | 40550.60 | 5.30 |
| GmASMT27 | Glyma.12G109800 | 363 | 40919.25 | 5.92 |
| GmASMT28 | Glyma.12G119600 | 326 | 36630.20 | 5.20 |
| GmASMT29 | Glyma.13G173300 | 365 | 40669.89 | 5.74 |
| GmASMT30 | Glyma.14G200800 | 283 | 31937.25 | 6.11 |
| GmASMT31 | Glyma.14G204900 | 358 | 40396.93 | 6.10 |
| GmASMT32 | Glyma.14G201000 | 242 | 27068.35 | 5.85 |
| GmASMT33 | Glyma.14G201100 | 358 | 40398.91 | 5.87 |
| GmASMT34 | Glyma.18G263700 | 378 | 41749.10 | 5.59 |
| GmASMT35 | Glyma.18G267500 | 359 | 40508.08 | 6.16 |
| GmASMT36 | Glyma.18G267800 | 354 | 39711.96 | 5.81 |
| GmASMT37 | Glyma.18G267900 | 352 | 40073.59 | 5.96 |
| GmASMT38 | Glyma.18G269600 | 355 | 40075.38 | 5.50 |
| GmASMT39 | Glyma.19G260700 | 372 | 41622.88 | 5.26 |
| GmASMT40 | Glyma.20G003500 | 370 | 41126.13 | 5.39 |
| GmASMT41 | Glyma.20G176100 | 366 | 40177.62 | 5.68 |
| GmASMT42 | Glyma.20G213500 | 354 | 39690.77 | 5.77 |
| GmASMT43 | Glyma.20G213600 | 354 | 39870.14 | 5.67 |
| GmASMT44 | Glyma.20G213700 | 354 | 39803.83 | 5.45 |

**Table S6.** Estimation of synonymous (Ks) and non-synonymous (Ka) substitution of duplicated *GmASMT* in *Glycine max*

| Gene | Ka | Ks | Ka/Ks | Time (MYA) | Duplication type |
| --- | --- | --- | --- | --- | --- |
| *GmASMT40/GmASMT19* | 0.029 | 0.160 | 0.179 | 13.090 | Segmental |
| GmASMT2/GmASMT1 | 0.030 | 0.081 | 0.363 | 6.675 | Tandem |
| GmASMT4/GmASMT3 | 0.017 | 0.217 | 0.078 | 17.806 | Segmental |
| GmASMT39/GmASMT11 | 0.381 | 1.697 | 0.225 | 139.106 | Segmental |
| GmASMT10/GmASMT9 | 0.791 | 2.528 | 0.313 | 207.240 | Tandem |
| GmASMT27/GmASMT8 | 0.047 | 0.251 | 0.189 | 20.557 | Segmental |
| GmASMT28/GmASMT5 | 0.065 | 0.183 | 0.354 | 14.974 | Segmental |
| GmASMT30/GmASMT31 | 0.014 | 0.053 | 0.270 | 4.377 | Tandem |
| GmASMT33/GmASMT32 | 0.016 | 0.013 | 1.276 | 1.033 | Tandem |
| GmASMT35/GmASMT12 | 0.124 | 0.218 | 0.570 | 17.853 | Segmental |
| GmASMT38/GmASMT15 | 0.058 | 0.185 | 0.311 | 15.178 | Segmental |
| GmASMT14/GmASMT13 | 0.240 | 0.424 | 0.567 | 34.726 | Tandem |
| GmASMT17/GmASMT16 | 0.003 | 0.038 | 0.091 | 3.086 | Tandem |
| GmASMT44/GmASMT21 | 0.021 | 0.174 | 0.123 | 14.265 | Segmental |

**Table S7**. List of genes that interacted with *GmASMT33* during seed developmental stages in soybean

| **Locus ID** | **Gene name** | **Function** |
| --- | --- | --- |
| LOC100793385 | 5'-3' exoribonuclease 4-like | MAPK signaling pathway, nucleic acid binding, exonuclease activity |
| LOC100796907 | ABC transporter family member 5 | ATP binding, ATPase activity, coupled to transmembrane movement of substances, |
| LOC100811613 | WAT1-related protein like | Auxin transport |
| LOC100820036 | protein altered seed germination 2 | negative regulation of fatty acid biosynthetic process, |
| LOC100782541 | abscisic acid 8'-hydroxylase 2 | sterol metabolic process, Carotenoid biosynthesis, Biosynthesis of secondary metabolites |
| LOC100793074 | chaperone protein, (chloroplast) | ATP binding, ATPase activity, protein metabolic process |
| LOC100815789 | chlorophyll a-b binding protein 215, | response to light stimulus, photosynthesis, light harvesting in photosystem I, protein-chromophore linkage |
| LOC100820014 | eukaryotic translation initiation factor 4B3-like | mRNA binding, translation initiation factor activity |
| LOC100818383 | fructose-bisphosphate aldolase 1 (chloroplast) | glycolytic process, fructose 1,6-bisphosphate metabolic process, Glycolysis / Gluconeogenesis, Metabolic pathways |
| LOC100812133 | gibberellin receptor GID1B-like | positive regulation of gibberellic acid mediated signaling pathway, raffinose family oligosaccharide biosynthetic process, floral organ morphogenesis, |
| LOC100796563 | lon protease homolog 2, peroxisomal | misfolded or incompletely synthesized protein catabolic process, protein targeting to peroxisome, protein processing, |
| LOC100793183 | mRNA-capping enzyme-like | 7-methylguanosine mRNA capping, |
| LOC100805393 | magnesium-protoporphyrin IX monomethyl ester (oxidative) cyclase, (chloroplast) | photosynthesis, chlorophyll biosynthetic process, Porphyrin metabolism, Metabolic pathways, |
| HIDH | 2-hydroxyisoflavanone dehydratase(HIDH) | isoflavonoid biosynthetic process, flavonoid biosynthetic process, isoflavonoid metabolic process, |
| FER2-1 | ferritin | iron ion transport, cellular iron ion homeostasis, intracellular sequestering of iron ion |
| GA20OX1 | gibberellin 20 oxidase 1 | response to light stimulus, gibberellin biosynthetic process, unidimensional cell growth, Diterpenoid biosynthesis |
| MAT1 | maturation-associated protein | response to stress, response to water, |
| LOC100805089 | myosin-11-like | endocytosis, vesicle organization, |
| LOC100783237 | nuclear poly(A) polymerase 4-like | mRNA polyadenylation, mRNA surveillance pathway |
| LOC100798485 | oxygen-evolving enhancer protein 1 (chloroplast) | photosystem II assembly, photosystem II stabilization, Photosynthesis, Metabolic pathways |
| LOC100820458 | oxygen-evolving enhancer protein 1 (chloroplast) | photosynthesis, photosystem II stabilization, Photosynthesis, Metabolic pathways |
| LOC100775408 | pentatricopeptide repeat-containing protein, (chloroplast) | nucleotide binding, nucleic acid binding, |
| LOC100794675 | Poly adenylate-binding protein RBP45 | nucleotide binding, nucleic acid binding, |
| LOC100788630 | protein aspartic protease in guard cell 2 | aspartic-type endopeptidase activity, |
| LOC100784482 | Rhodanese-like domain-containing protein 4 (chloroplast) | chitin catabolic process, |
| LOC100791165 | splicing factor, suppressor of white-apricot homolog | mRNA 5'-splice site recognition, |
| SRC1 | src1 protein | metal ion binding, |
| LOC100800779 | Transcription factor TCP20-like | Transcription factor, TCP, Transcription factor TCP subgroup, |
| LOC100807306 | Transmembrane 9 superfamily member 12-like | integral component of membrane, |
| LOC100776447 | Ubiquitin domain-containing protein DSK2a-like | Ubiquitin, Heat shock chaperonin-binding, UBA-like, Ubiquilin, Ubiquitin-associated/translation elongation factor EF1B, |
| LOC100500275 | Uncharacterized | Rubber elongation factor, |
| LOC100786263 | Uncharacterized | integral component of membrane, |
| LOC100793415 | Uncharacterized | Calycin-like, |
| LOC100500135 | Zinc finger CCHC domain-containing protein | negative regulation of transcription, DNA-templated, |
| LOC100788280 | uncharacterized zinc finger CCHC domain-containing protein-like | negative regulation of transcription, DNA-templated, |
| LOC100794120 | Vesicle-associated protein 4-2-like | endoplasmic reticulum membrane, |
| LOC100817967 | Heme-binding domain-containing protein |  |
| IMAT1 | Isoflavone malonyltransferase IMaT1 | transferase activity, transferring acyl groups other than amino-acyl groups, |
| LOC100787464 | Uncharacterized | mRNA processing, |
| LOC112998375 | Uncharacterized | RNA processing, |
| SUBI 2 | Ubiquitin-like | Polyubiquitin |

**Table S8**. List of genes that interacted with *GmASMT44* during heat and drought stress in soybean

| **Locus ID** | **Gene name** | **Function** |
| --- | --- | --- |
| LOC100813126 | AAA-ATPase ASD, mitochondrial | integral component of membrane |
| LOC100787917 | AAA-ATPase | integral component of membrane, |
| LOC100806805 | BON1-associated protein 2 | C2 calcium-dependent membrane targeting |
| LOC100305356 | CC-NBS-LRR class disease resistance protein | defense response, |
| LOC100800161 | E3 ubiquitin-protein ligase RGLG5 | Zinc finger, RING-type, |
| LOC100819737 | G-type lectin S-receptor-like serine/threonine-protein kinase | Protein kinase, catalytic domain, Serine-threonine/tyrosine-protein kinase catalytic domain |
| LOC100794508 | G-type lectin S-receptor-like serine/threonine-protein kinase CES101 | protein serine/threonine kinase activity, ATP binding, |
| LOC100794505 | Leaf rust 10 disease-resistance locus receptor-like protein kinase-like 2.4 | Protein kinase, catalytic domain, Serine-threonine/tyrosine-protein kinase catalytic domain, |
| LOC102665964 | MDIS1-interacting receptor like kinase 2 | protein autophosphorylation |
| NRP-A | N-rich protein | response to water deprivation, response to endoplasmic reticulum stress, stress response |
| NAC6 | NAC domain protein NAC6 | Stress response, Transcription, Transcription regulation, |
| LOC100816770 | NAC domain-containing protein | regulation of transcription, DNA-templated, |
| LOC100812364 | RING-H2 finger protein ATL16 | protein ubiquitination, |
| LOC100795816 | U-box domain-containing protein 19 | ubiquitin-protein transferase activity, |
| LOC100799795 | U-box domain-containing protein 54 | UspA, Rossmann-like alpha/beta/alpha sandwich fold, |
| LOC100781197 | UDP-glucose 6-dehydrogenase 1 | glycosaminoglycan biosynthetic process, UDP-glucuronate biosynthetic process, pentose and glucuronate interconversions |
| WRKY6 | WRKY transcription factor 6 | transcription factor activity, sequence-specific DNA binding |
| LOC100813788 | auxin-responsive protein IAA13-like | regulation of transcription, DNA-templated, auxin-activated signaling pathway, plant hormone signal transduction, |
| LOC100802677 | calcium-binding protein KIC | calcium ion binding, |
| SCAM-4 | calmodulin | calcium ion binding, plant-pathogen interaction |
| LOC100819818 | calmodulin-binding receptor-like cytoplasmic kinase 2 | protein phosphorylation |
| LOC100776652 | calreticulin-3 | protein folding, Protein processing in endoplasmic reticulum, Phagosome, |
| CRK21 | cysteine-rich receptor-like protein kinase | protein phosphorylation |
| LOC100803576 | cytochrome b561 | Electron transport |
| LOC100818119 | cytochrome b561 | Electron transport |
| LOC100780491 | derlin-1.1 | ubiquitin-dependent ERAD pathway, protein processing in endoplasmic reticulum |
| LOC100814688 | disease resistance protein RPP13 | defense response |
| LOC100795901 | early nodulin-like protein 2-like | anchored component of plasma membrane, electron carrier activity |
| LOC100811093 | epsin-3 | endocytosis |
| LOC100792129 | galactinol synthase 2 | galactose metabolic process |
| LOC100306170 | heavy-metal-associated domain-containing protein | metal ion binding |
| LOC547566 | indole-3-acetic acid induced protein ARG-2 homolog | Late embryogenesis abundant protein |
| LOC100305427 | lectin-like receptor kinase | defense response to biotic |
| LOC100803042 | lysM domain receptor-like kinase 3 | protein serine/threonine kinase activity, ATP binding |
| LOC100782377 | metalloendoproteinase 5-MMP | extracellular matrix organization, collagen catabolic process |
| LOC106795478 | nudix hydrolase 2 | NADH pyrophosphatase activity, metal ion binding, ADP-ribose diphosphatase activity, NAD binding, |
| PEPC4 | phosphoenolpyruvate carboxylase | tricarboxylic acid cycle, carbon fixation, Pyruvate metabolism, Carbon fixation |
| LOC100796836 | probable WRKY transcription factor 50 | transcription factor activity, sequence-specific DNA binding |
| LOC100791756 | probable calcium-binding protein CML46 | hormone-mediated signaling pathway |
| LOC100797934 | probable protein phosphatase 2C 25 | protein serine/threonine phosphatase activity |
| LOC100819703 | protein EDS1L | lipid metabolic process, defense response |
| LOC100818548 | putative glycolipid transfer protein | ceramide 1-phosphate binding and transporter activity |
| LOC102669457 | rust resistance kinase Lr10 | protein kinase activity, ATP binding |
| LOC100792931 | scarecrow-like protein 13-like | transcription factor activity, sequence-specific DNA binding |
| LOC100500035 | uncharacterized | **_** |
| LOC100785051 | uncharacterized | **_** |
| LOC100810460 | uncharacterized | **_** |
| LOC100819406 | uncharacterized | **_** |

**Table S9**. List of genes that interacted with *GmASMT17* during aphid infections in soybean

| **Locus ID** | **Gene name** | **Function** |
| --- | --- | --- |
| LOC102667868 | 1-aminocyclopropane-1-carboxylate oxidase homolog 12 | oxidoreductase activity, metal ion binding, |
| NDR1b | CC-NB-LRR resistance (R) protein | integral component of membrane, anchored component of plasma membrane, |
| LOC100527510 | CCT motif and tify domain-containing protein | response to wounding, regulation of defense response, regulation of jasmonic acid mediated signaling pathway |
| LOC100776993 | IQ domain-containing protein IQM1() | Nuclear protein |
| MYB124 | MYB transcription factor MYB124 | regulation of transcription |
| LOC100784960 | NAC domain-containing protein 90 | regulation of transcription |
| LOC100808618 | NAC domain-containing protein 90 | regulation of transcription |
| MYB12A | R2R3-type MYB transcription factor MYB12a | regulation of transcription |
| LOC100793323 | SNAP25 homologous protein SNAP33-like | protein transport |
| LOC100794340 | U-box domain-containing protein 21 | ubiquitin-protein transferase activity |
| WRKY47 | WRKY transcription factor 47 | regulation of transcription |
| ALDH3H2 | aldehyde dehydrogenase family 3 member H2 | cellular aldehyde metabolic process |
| AOS1 | allene oxide synthase | jasmonic acid biosynthetic process |
| AOS2 | allene oxide synthase | jasmonic acid biosynthetic process |
| LOC100794597 | auxin-induced protein 6B-like | auxin-induced protein 6B-like(LOC100794597) |
| LOC100775893 | berberine bridge enzyme-like 21 | oxidoreductase activity, FAD binding |
| LOC100780083 | calcium uniporter protein 6, mitochondrial | mitochondrial calcium uptake, mitochondrial calcium ion homeostasis |
| LOC100808399 | calcium-dependent protein kinase 32 | peptidyl-serine phosphorylation, intracellular signal transduction, protein autophosphorylation |
| SCAM-4 | calmodulin | calcium ion binding, |
| LOC100817351 | calmodulin-like | calcium ion binding, |
| CHS9 | chalcone synthase 9 | flavonoid biosynthetic process |
| LOC100802250 | cyanogenic beta-glucosidase | carbohydrate metabolic process, |
| LOC100811368 | cysteine-rich and transmembrane domain-containing protein B | plasma membrane, integral component of membrane |
| LOC100793366 | ethylene-responsive transcription factor 13 | transcription factor |
| LOC100788567 | ethylene-responsive transcription factor ERF022 | transcription factor |
| GA2OX8 | gibberellin 2-beta-dioxygenase 8 | Diterpenoid biosynthesis, Biosynthesis of secondary metabolites, |
| LOC100777511 | glutamyl-tRNA reductase 2, chloroplastic | protoporphyrinogen IX biosynthetic process, |
| LOC100805897 | glycosyltransferase BC10 | integral component of membrane, |
| HSF-20 | heat stress transcription factor 20 | regulation of transcription from RNA polymerase II promoter, |
| LOC100776617 | heavy metal-associated isoprenylated plant protein 47 | metal ion binding, |
| LOC100781793 | lysine-rich arabinogalactan protein 19 | Signal transduction |
| LOC100798607 | mitogen-activated protein kinase kinase kinase 2-like | NADH pyrophosphatase activity, metal ion binding, ADP-ribose diphosphatase activity, |
| LOC100527186 | nudix hydrolase family protein | nucleus, cytoplasm, |
| FAD7-2 | omega-3 fatty acid desaturase, chloroplastic | lipid metabolic process, unsaturated fatty acid biosynthetic process |
| LOC100817853 | oxalate--CoA ligase | fatty acid metabolic process, |
| LOC100778514 | phospholipase A1-Ibeta2, chloroplastic | lipid metabolic process, |
| LOC100819591 | phospholipase A1-Ibeta2, chloroplastic | lipid metabolic process, |
| LOC100778648 | probable F-box protein | integral component of membrane, |
| LOC100806989 | probable inactive patatin-like protein 9 | lipid catabolic process, |
| LOC100776488 | probable indole-3-acetic acid-amido synthetase GH3.1 | Plant hormone signal transduction, |
| LOC100813810 | probable protein phosphatase 2C 63 | Protein phosphatase 2C |
| LOC100802394 | probable protein phosphatase 2C 78 | Protein phosphatase 2C |
| LOC100777578 | probable serine/threonine-protein kinase PIX13 | protein serine/threonine kinase activity |
| LOC100816060 | probable serine/threonine-protein kinase WNK11 | protein phosphorylation, intracellular signal transduction, |
| LOC100782104 | probable xyloglucan endotransglucosylase/hydrolase protein 23 | xyloglucan metabolic process, cell wall biogenesis, cell wall organization, |
| LOC100784249 | probable xyloglucan endotransglucosylase/hydrolase protein 23 | cellular glucan metabolic process, |
| LOC100801634 | probable xyloglucan endotransglucosylase/hydrolase protein 23 | xyloglucan metabolic process, cell wall biogenesis/degradation, |
| LOC100817983 | protein LURP-one-related 10 | LURP1-like domain, Tubby C-terminal-like domain, |
| LOC100802800 | protein TIFY 10A-like | response to wounding, regulation of defense response |
| LOC100799869 | putative F-box protein PP2-B12 | F-box domain, cyclin-like, Phloem protein 2-like, |
| LOC100815307 | putative PAR1 protein | Signal transduction |
| LOC100815307 | putative PAR1 protein() | N-acetylglucosamine metabolic process |
| LOC100777057 | putative calcium-bindingprotein CML19 | calcium ion binding, |
| LOC100798531 | putative calcium-binding protein CML19 | calcium ion binding, |
| LOC100786639 | Putative serine/threonine-protein kinase-like protein CCR3 | integral component of membrane, |
| LOC100306152 | response regulator ARR6-like | phosphorelay signal transduction system, |
| LOC100781099 | rust resistance kinase Lr10 | integral component of membrane, |
| LOC100806180 | serpin-ZX | negative regulation of endopeptidase activity, |
| LOC100791118 | signaling peptide TAXIMIN 2 | integral component of membrane, |
| LOC100820571 | signaling peptide TAXIMIN 2 | integral component of membrane, |
| LOC100817788 | tetraspanin-8 | plasma membrane, plasmodesma, integral component of membrane, |
| LOC100781727 | transcription factor MYB77 | regulation of transcription, |
| LOC100777932 | transcription factor MYB78 | regulation of transcription |
| SCAM-4 | calmodulin like | Response to calcium ion binding, pathogen or NaCl stresses |
| LOC100813871 | zinc finger protein ZAT12 | Zinc finger C2H2-type/integrase DNA-binding domain |
| LOC100306332 | uncharacterized protein | response to wounding, regulation of defense response |
| LOC100805850 | putative beta-1,4-mannosyl-glycoprotein 4-beta-N-acetylglucosaminyltransferase | N-acetylglucosamine metabolic process, protein N-linked glycosylation, |
| LOC100306678 | uncharacterized protein |  |
| LOC100500311 | uncharacterized protein |  |
| LOC100776077 | uncharacterized protein |  |
| LOC100783731 | uncharacterized protein |  |
| LOC100807334 | uncharacterized protein |  |
| LOC102662202 | uncharacterized protein |  |
| LOC106795509 | uncharacterized protein |  |
| LOC100799846 | uncharacterized protein |  |
| LOC100786682 | uncharacterized protein |  |
| LOC100804600 | uncharacterized protein |  |

**Table S10**. List of genes that interacted with *GmASMT7* during soybean cyst nematode infections in soybean

| **Locus ID** | **Gene name** | **Function** |
| --- | --- | --- |
| LOC100809841 | CBS domain-containing protein CBSCBSPB3-like | integral component of membrane, regulate redox homeostasis |
| EKN | EKN protein | basic and acidic residues containing protein |
| LOC100815109 | F-box protein-like | proteasome-mediated ubiquitin-dependent protein catabolic process, |
| LOC102663618 | F-box/kelch-repeat protein-like | F-box domain, cyclin-like, F-box associated interaction domain, |
| LOC100786055 | IQ domain-containing protein | nuclear protein |
| LOC100791383 | NEP1-interacting protein 2 | integral component of membrane |
| LOC100814384 | RING-H2 finger protein ATL34-like | integral component of membrane, Signal, Transmembrane |
| LOC100801890 | TMV resistance protein N-like | defense response, signal transduction, |
| LOC100775205 | bax inhibitor 1 | integral component of membrane, Bax inhibitor 1-related |
| LOC100782913 | casein kinase 1-like protein 3 | endocytosis, peptidyl-serine phosphorylation |
| LOC100780817 | cellulose synthase-like protein B5 | plant-type primary cell wall biogenesis, cellulose biosynthetic process |
| LOC100801936 | cytochrome P450 | monooxygenase activity, iron ion binding, oxidoreductase activity, acting on paired donors with incorporation or reduction of molecular oxygen |
| LOC100809551 | Cytosolic sulfotransferase 15 | sulfation, |
| LOC102665316 | embryo-specific protein ATS3B | embryo-specific 3 cytoplasm protein |
| GS1-GAMMA | gamma glutamine synthetase | amino acid biosynthesis |
| LOC100793109 | nodulin Mt N21-like transporter family protein | plasma membrane, integral component of membrane |
| N-23 | nodulin-23 | nodulation, |
| LOC100808832 | potassium channel AKT1 | integral component of membrane, voltage-gated potassium channel activity |
| LOC100807538 | probable 2-isopropylmalate synthase | biosynthesis of amino acids |
| N-56 | probable 2-isopropylmalate synthase | biosynthesis of amino acids |
| LOC100777694 | probable amino-acid acetyltransferase NAGS2, chloroplastic | arginine biosynthetic process |
| LOC100813954 | probable polyol transporter 6 | transmembrane transporter activity |
| LOC100819870 | probable protein phosphatase 2C 38 | metal ion binding, manganese/magnesium aspartate binding site |
| FWL3 | protein FW2.2-like 3 | uncharacterised protein family Cys-rich |
| LOC100791180 | NRT1/ PTR family protein 1.2 | transmembrane transporter activity |
| LOC100789214 | protein NRT1/ PTR FAMILY 5.1 | transmembrane transporter activity |
| LOC100805054 | protein NRT1/ PTR FAMILY 5.1 | transmembrane transporter activity |
| LOC102662472 | putative F-box/FBD/LRR-repeat protein | F-box domain, cyclin-like |
| LOC100527376 | uncharacterized protein |  |
| LOC100527523 | uncharacterized protein |  |
| LOC100812465 | uncharacterized protein |  |
| LOC100777865 | uncharacterized protein |  |
| LOC100811594 | uncharacterized protein |  |
| LOC121173076 | uncharacterized protein |  |


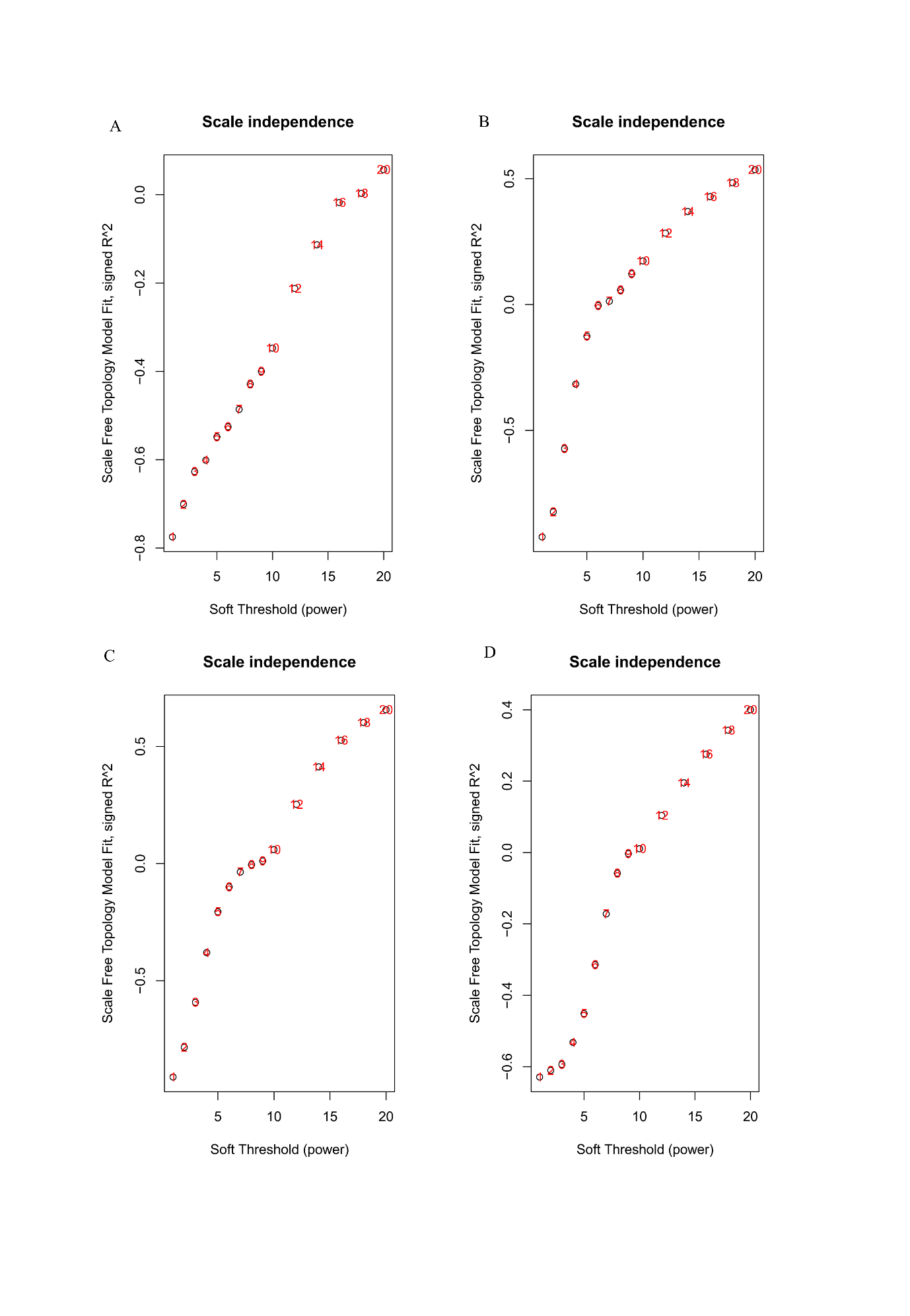


**Figure S1**. Determination of soft threshold power used for WGCNA in **A** Embryo development (β = 10), **B** abiotic stress (β = 14), **C** Aphid infection (β = 14), and **D** SCN infection (β = 14).


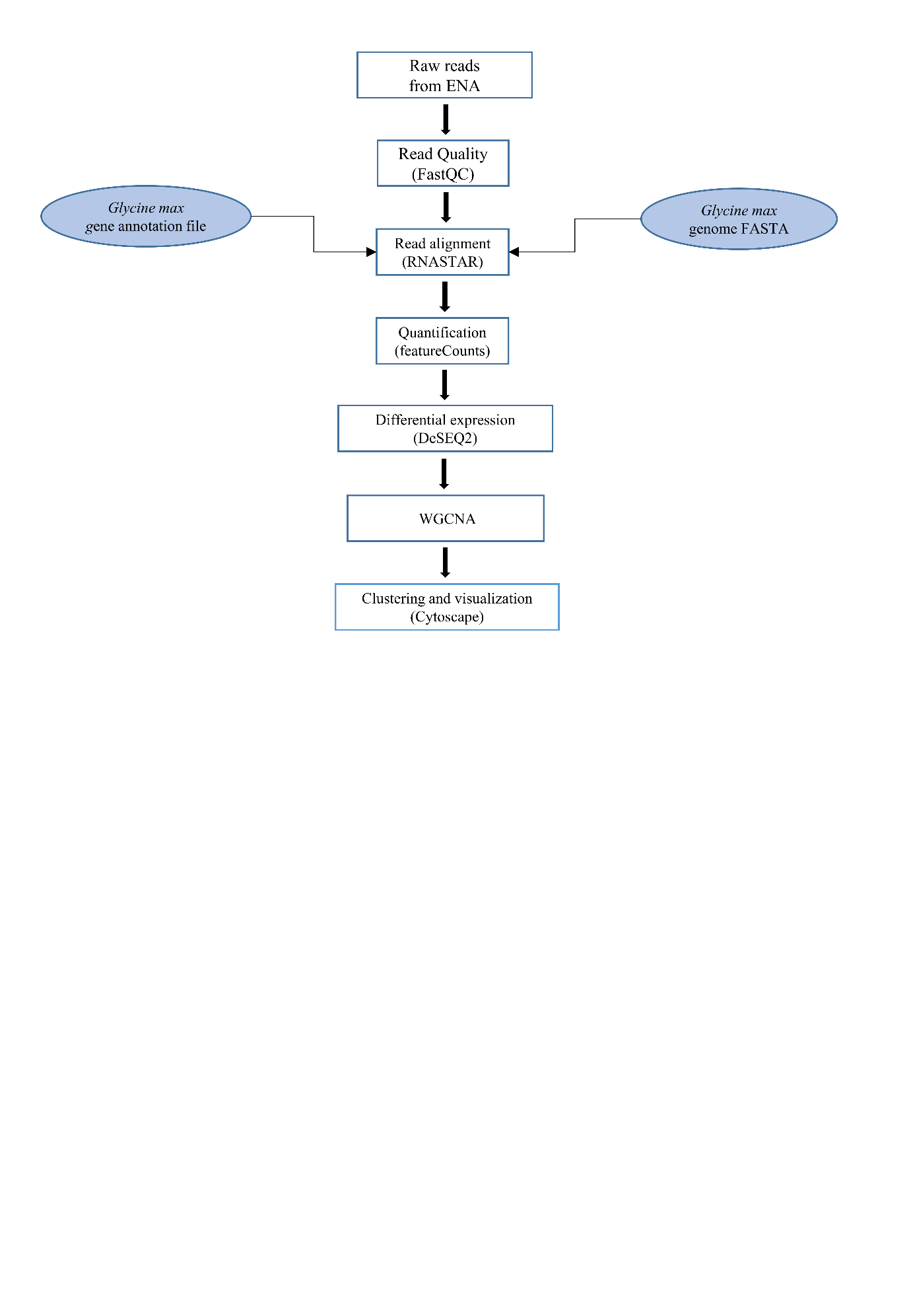
**Figure S2**. Workflow for the RNA-seq dataset analysis for embryo development, biotic and abiotic stress.


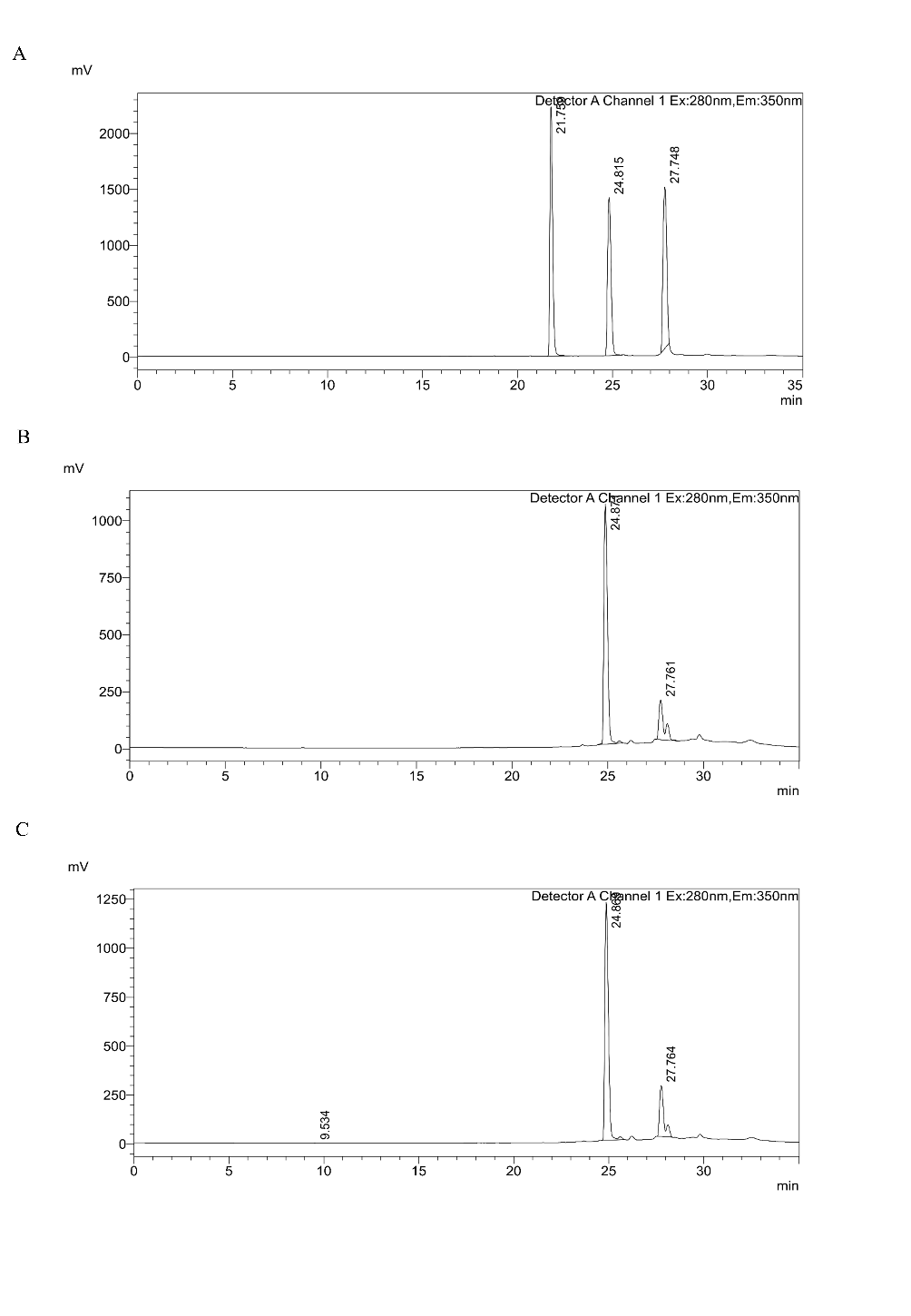


**Figure S3.** The HPLC chromatogram for Mel enzyme assay **A** Standards of SER (RT = 21.7), N-Acetylserotonin (RT = 24.8), and MEL (RT = 27.7) **B** GmASMT33, and **C** GmASMT44.


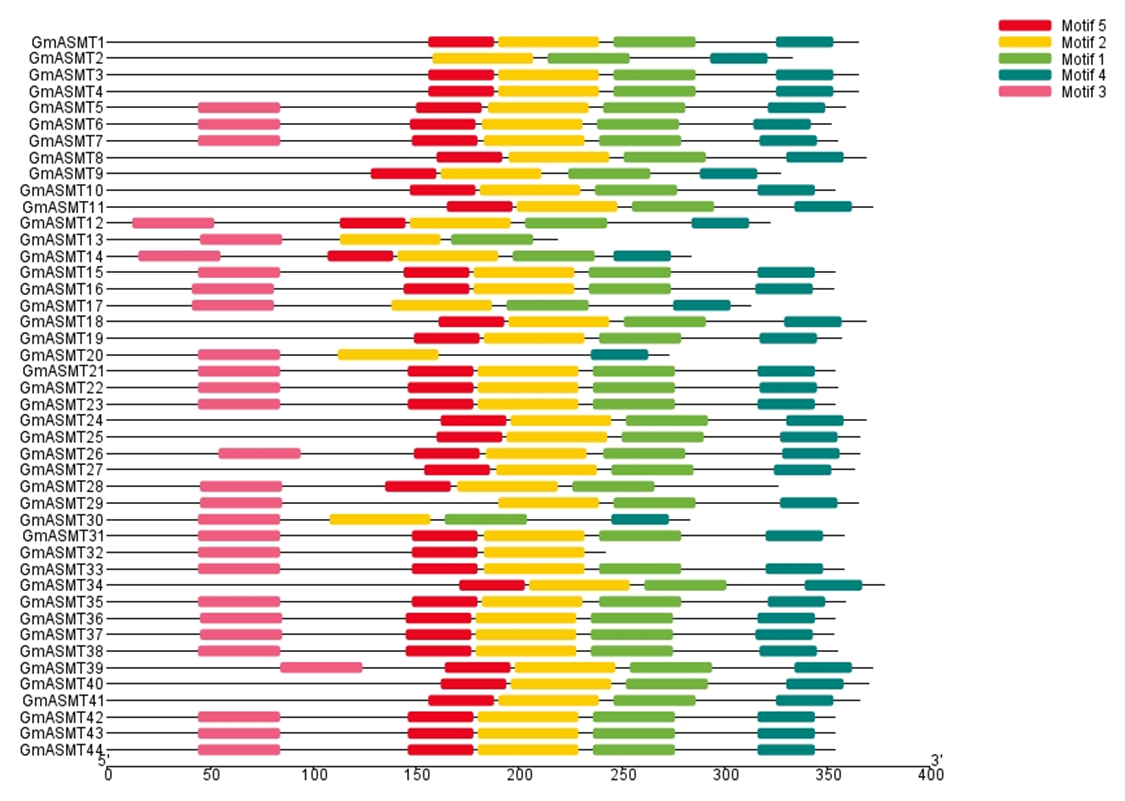


**Figure S4.** The motif compositions of *GmASMTs*: The width of grey bars at the bottom display relative lengths of *GmASMTs*. Different colour boxes represent different motifs present in *GmASMTs.* Motif 1, 3, and 5, encodes for O-methyltransferase, whereas motif 2 and 5 encode for dimerization.


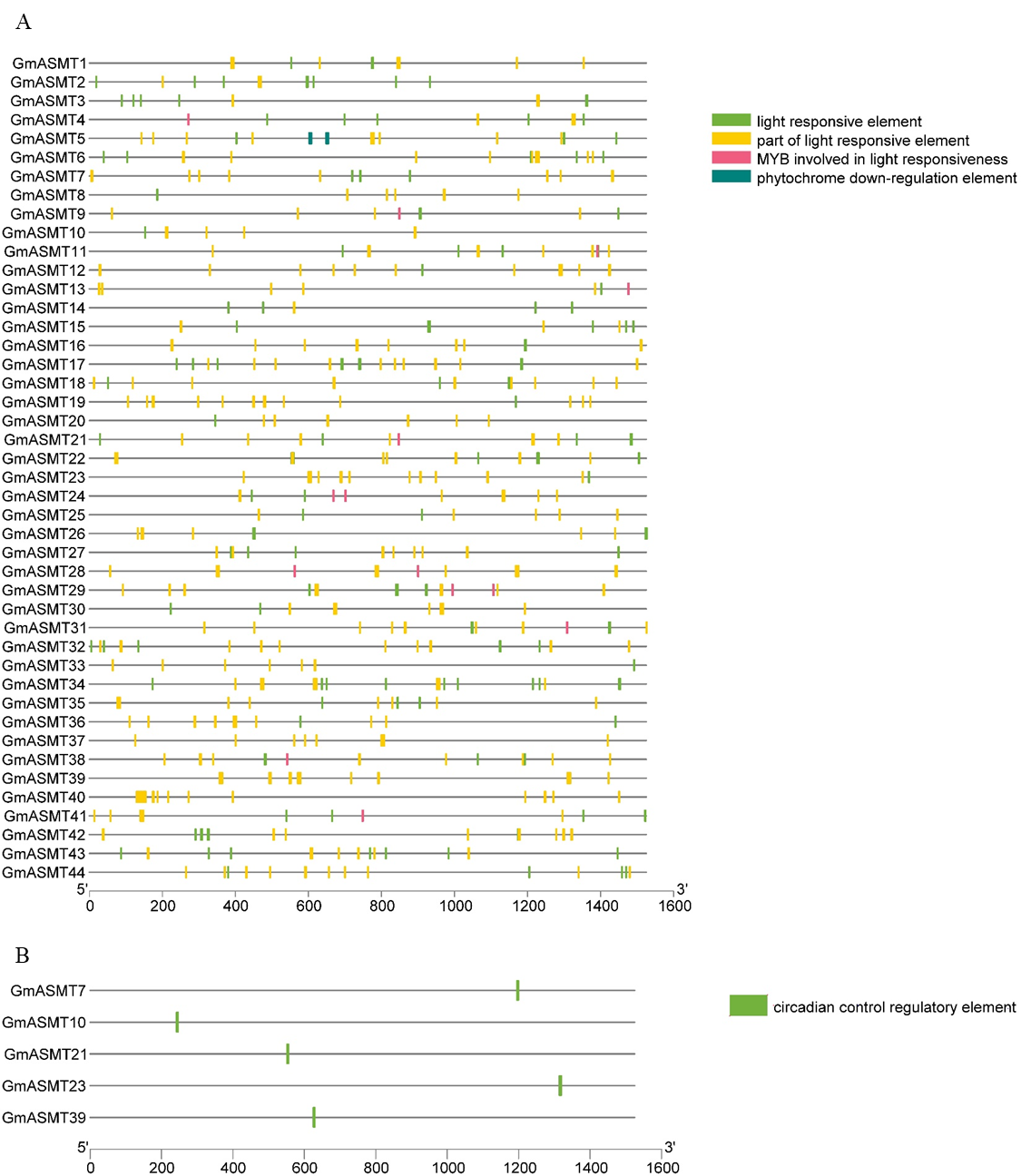


**Figure S5.** Distribution of **A** Light **B** circadian cis-elements in the promoter sequences of putative GmASMTs. light, partial light, MYB involved in light, phytochrome down-regulation responsive elements, and circadian regulatory element are represented by the different colours on individual gene sequences as indicated in figure key on the right side.


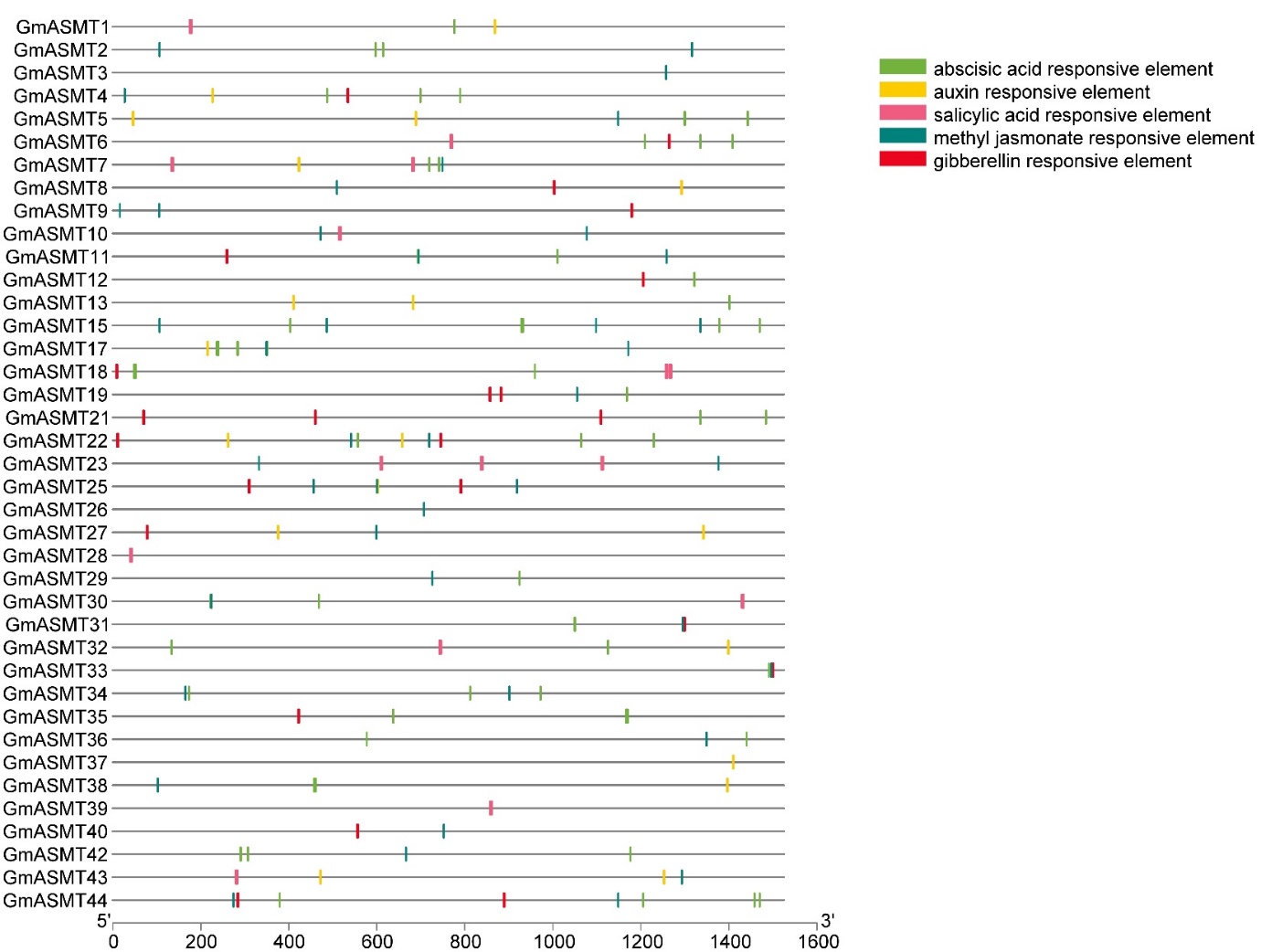


**Figure S6**. Distribution of major plant growth hormone regulatory elements on the promoter sequences of putative *GmASMTs*. abscisic acid, auxin, salicylic acid, methyl jasmonate, and gibberellic acid-responsive elements are represented by the different colours on individual gene sequences as indicated in the figure key on the right side.


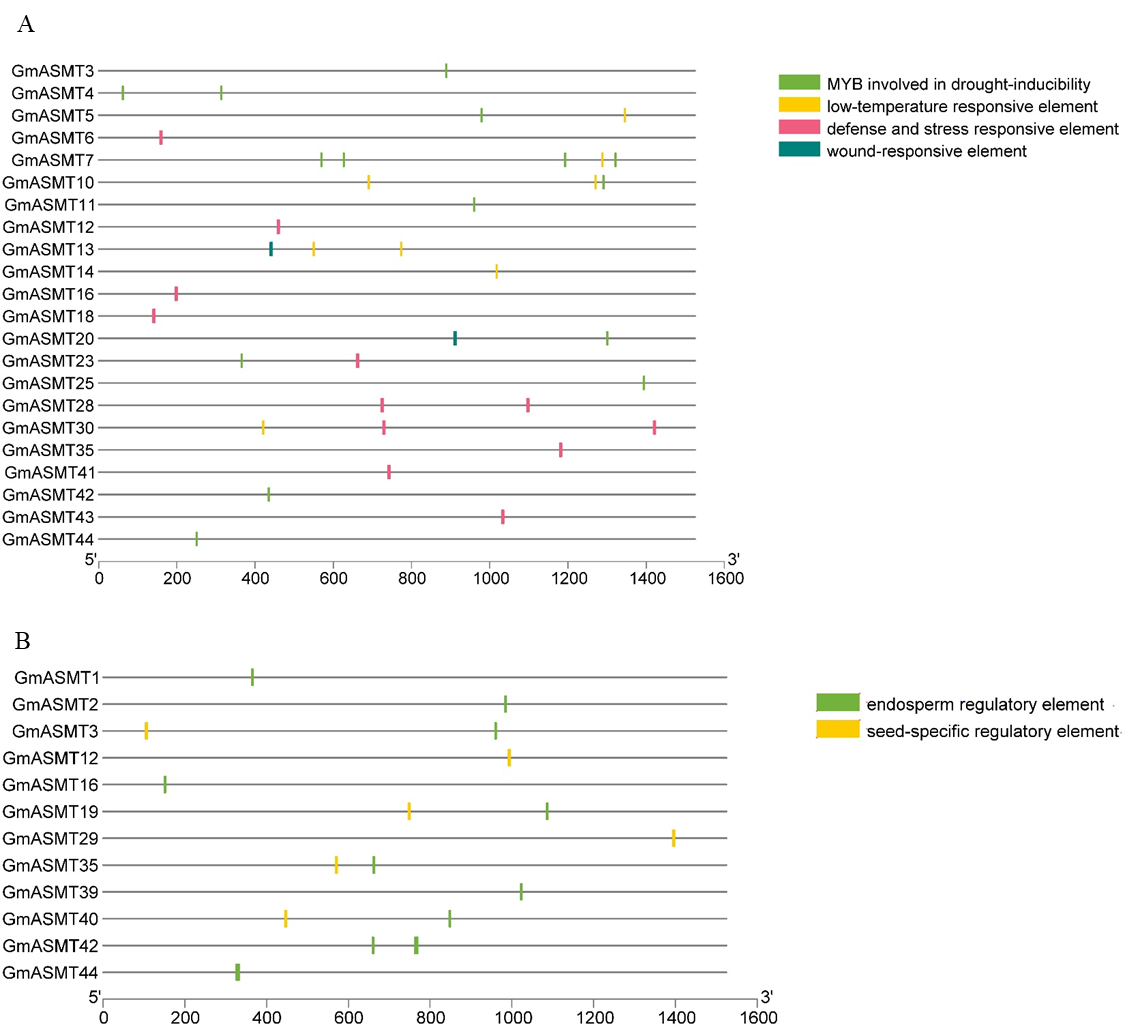


**Figure S7**. **A** Distribution of major plant stress-responsive elements in the promoter sequences of 25 putative *GmASMTs*. MYB is involved in drought low temperature, defense, and wound-responsive elements. **B** Distribution of endosperm and seed-specific regulatory elements in the promoter sequences of 12 putative *GmASMTs.* The regulatory elements are represented by the different colours on individual gene sequences, as indicated in the figure key on the right side.


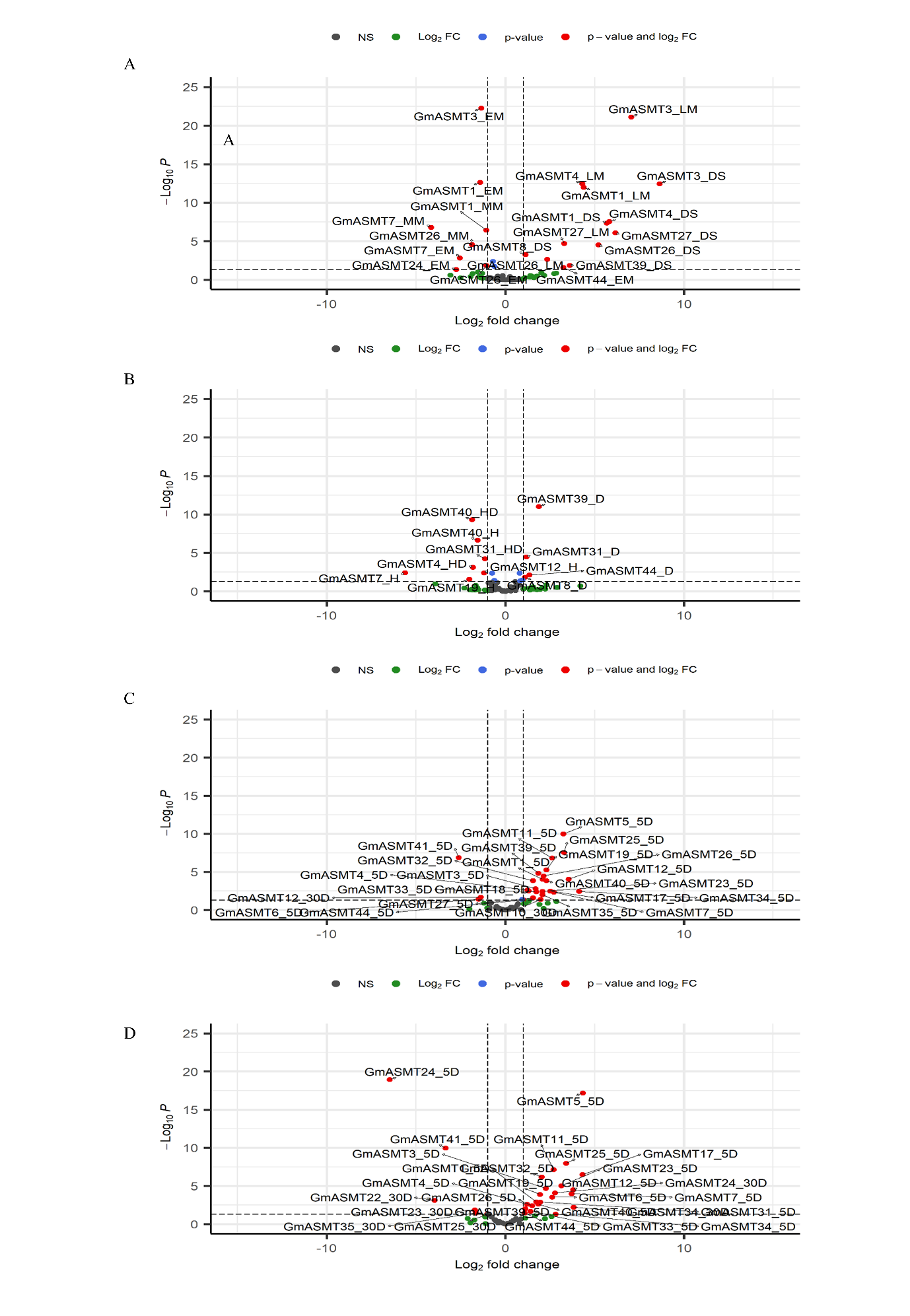


**Figure S8.** The volcano plot for **A** embryo development **B** abiotic stress **C** Aphid infections and **D** SCN infections. The genes that meet both log fold change (±1.5) and Padj cutoff (<0.05) of the datasets are highlighted in the red color in the volcano plot.


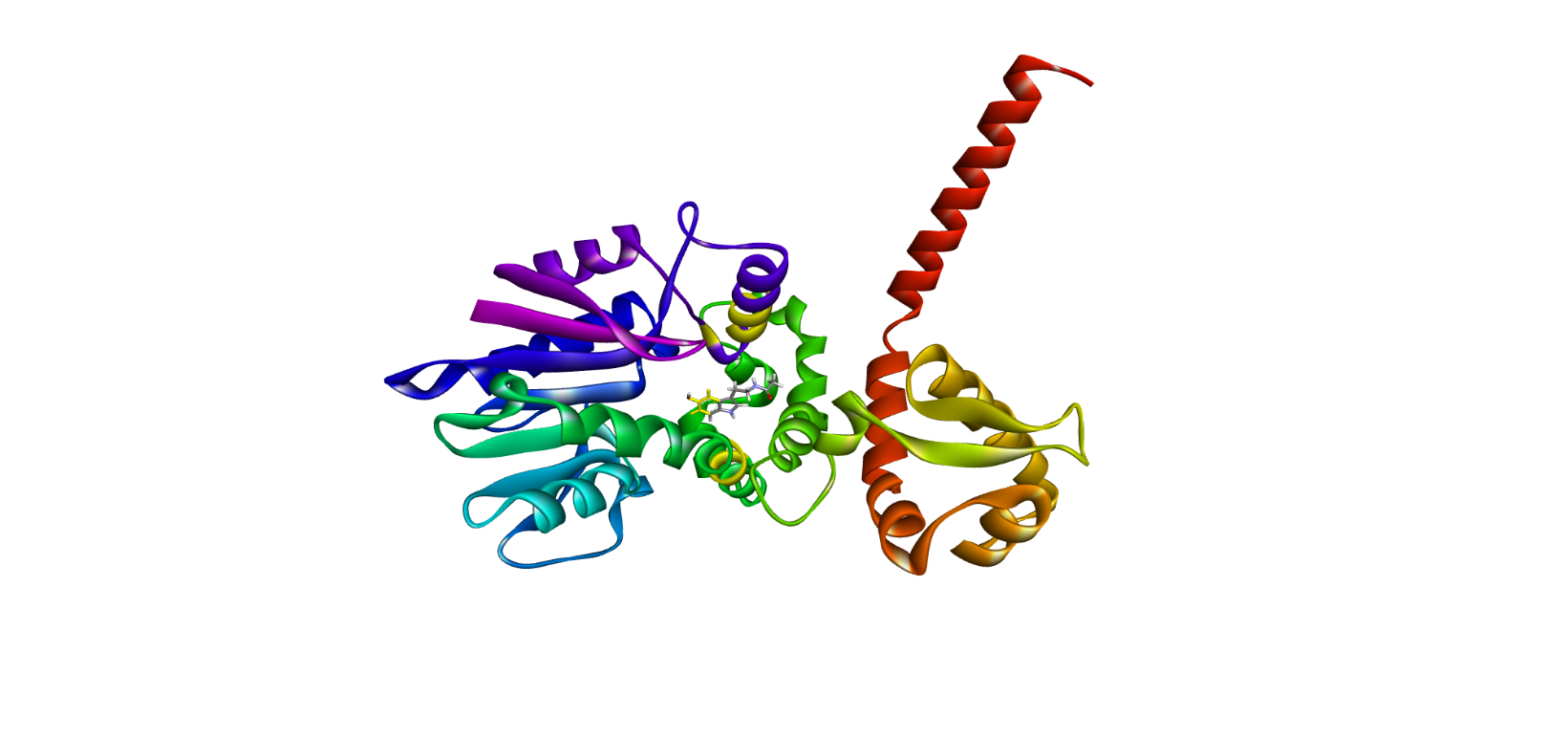

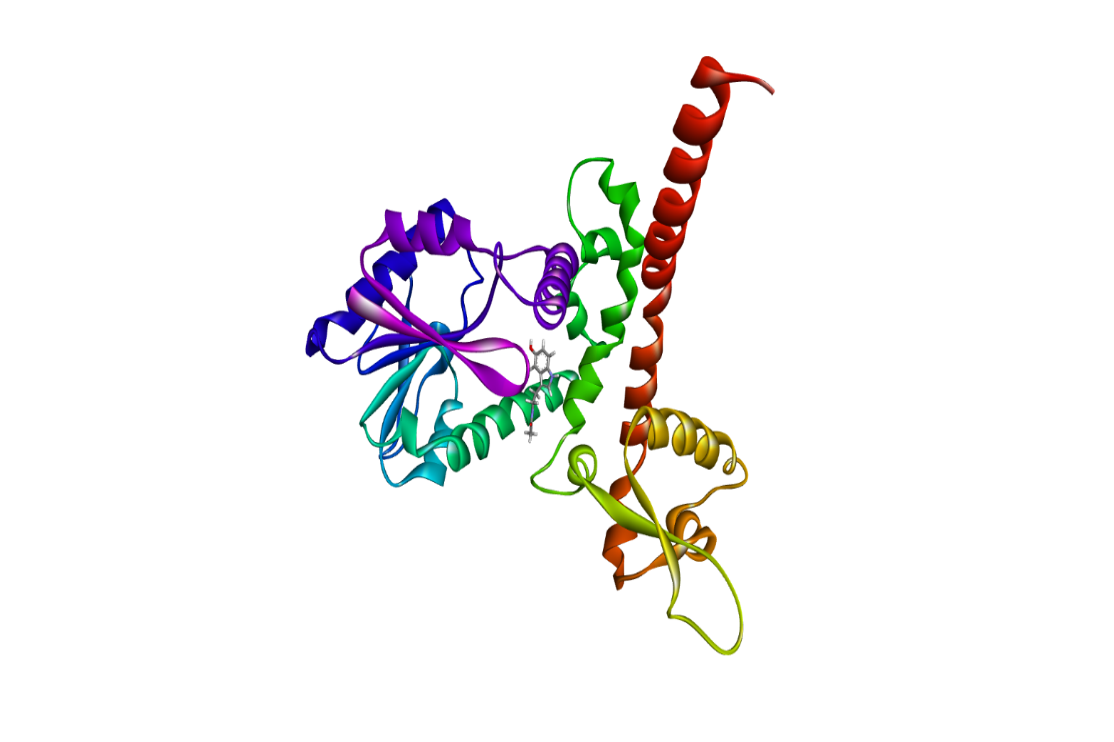


A

B


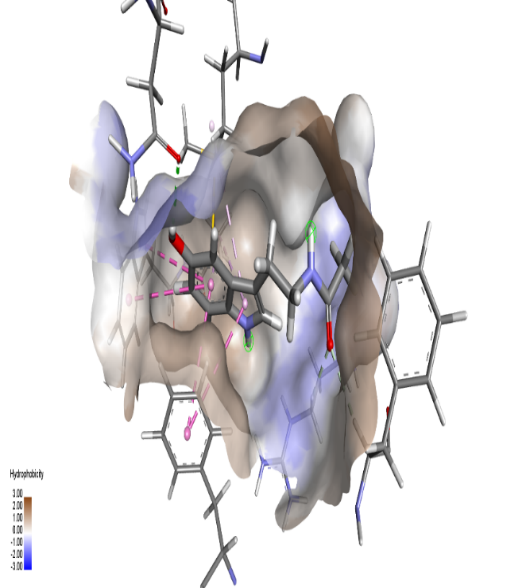

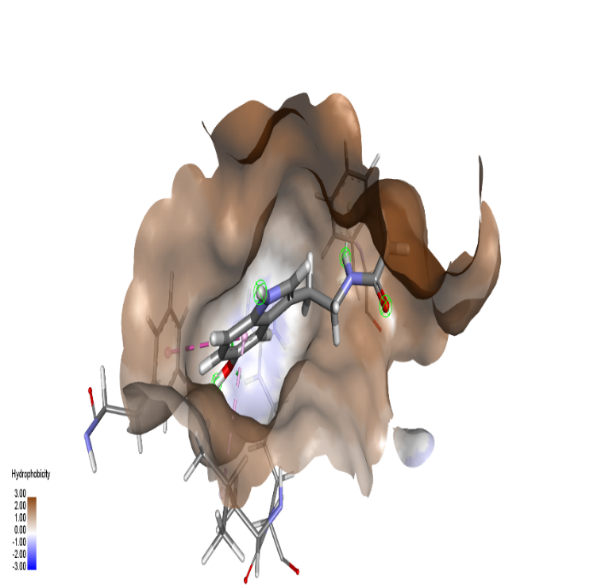


C

D


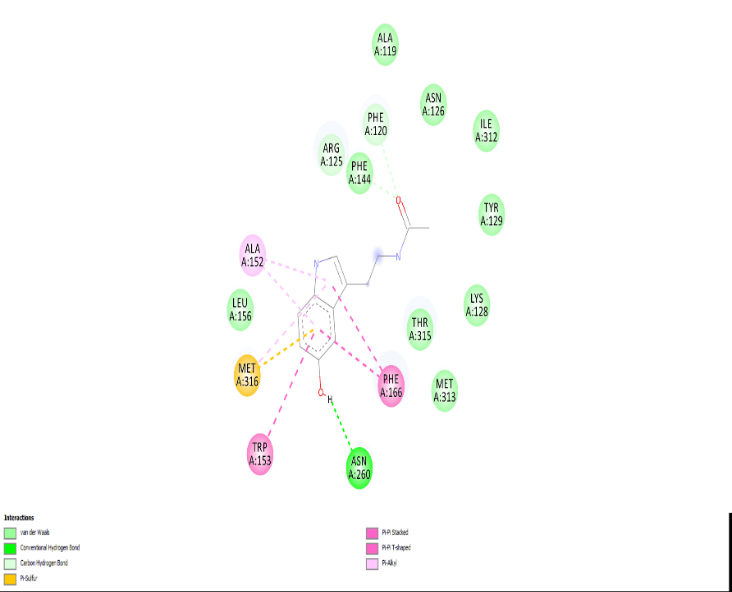

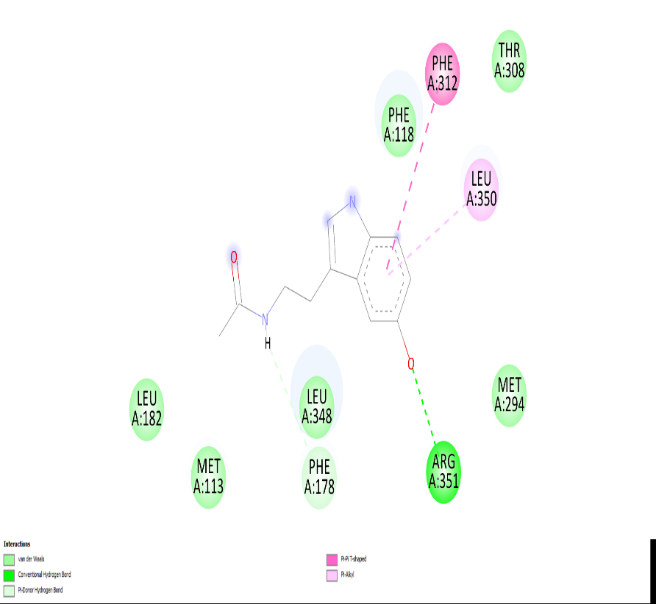


E

F

**Figure S9**. The 3D modeling and molecular docking analysis of *GmASMT* protein with N-acetyl serotonin. Three-dimensional model of **A** *GmASMT33* and **B** *GmASMT44*. Protein-ligand molecular docking analysis **C** *GmASMT33* and **D** *GmASMT44* protein with N acetyl serotonin. The 2D plot of docked N-acetylserotonin with **E** *GmASMT33* and **F** *GmASMT44*.
